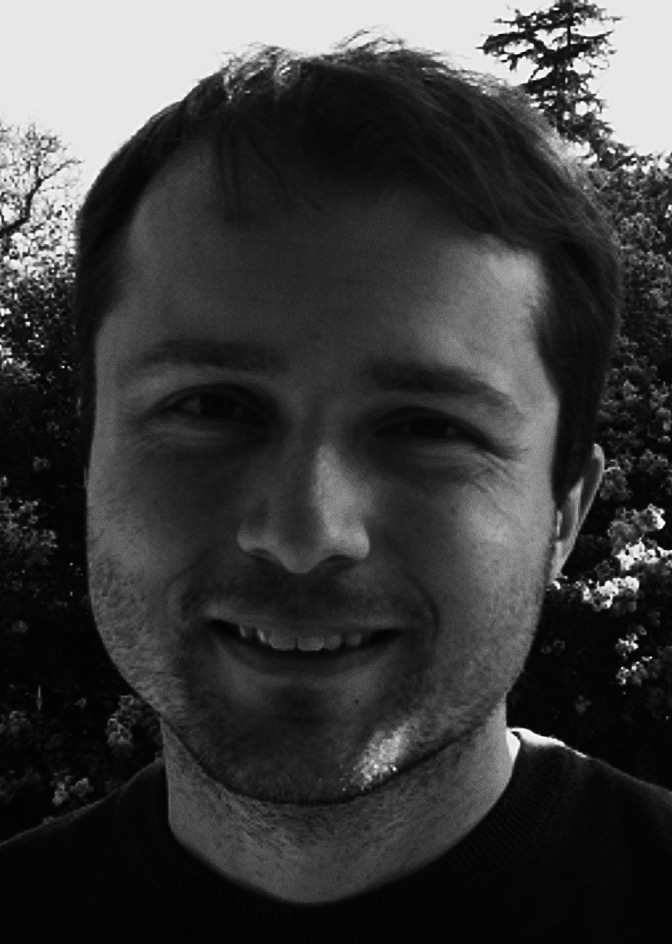# Recent Progress and Emerging Technologies towards a Sustainable Synthesis of Sulfones

**DOI:** 10.1002/cssc.202101635

**Published:** 2021-10-13

**Authors:** Shuai Liang, Kamil Hofman, Marius Friedrich, Julian Keller, Georg Manolikakes

**Affiliations:** ^1^ Department of Medicinal Chemistry, School of Pharmacy Qingdao University Medical College No.1 Ningde Road 266073 Qingdao P. R. China; ^2^ Department of Chemistry TU Kaiserslautern Erwin-Schrödinger-Str. Geb. 54 D-67663 Kaiserslautern Germany

**Keywords:** electrochemistry, photocatalysis, sulfone, sulfur dioxide, sustainable chemistry

## Abstract

Sulfones play a pivotal role in modern organic chemistry. They are highly versatile building blocks and find various applications as drugs, agrochemicals, or functional materials. Therefore, sustainable access to this class of molecules is of great interest. Herein, the goal was to provide a summary on recent developments in the field of sustainable sulfone synthesis. Advances and existing limitations in traditional approaches towards sulfones were reviewed on selected examples. Furthermore, novel emerging technologies for a more sustainable sulfone synthesis and future directions were discussed.

## Graphical abstract

**SO(2) Green**: This Review covers recent developments and advances towards a green synthesis of the sulfone (R−SO_2_−R’) functionality. Improvements of classical methods and emerging technologies, such as photo‐ and electrochemical methods or the direct fixation of SO_2_, are covered and evaluated in terms of their sustainability.

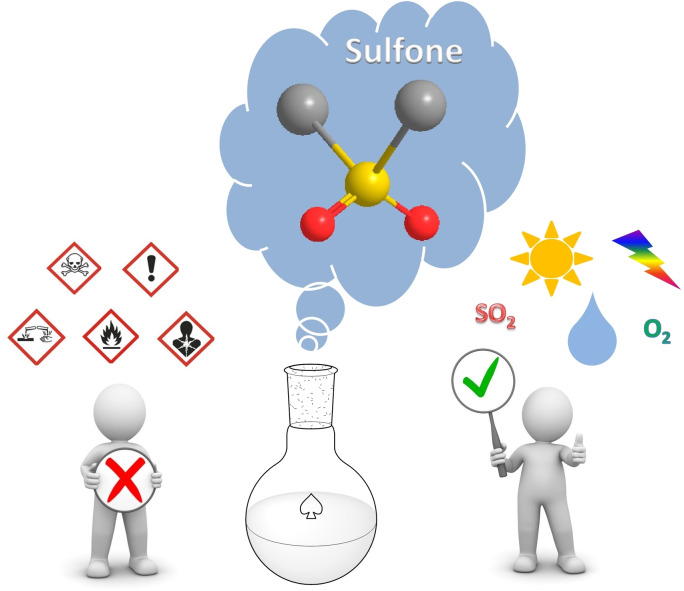

## 1 Introduction

**Figure 1:**
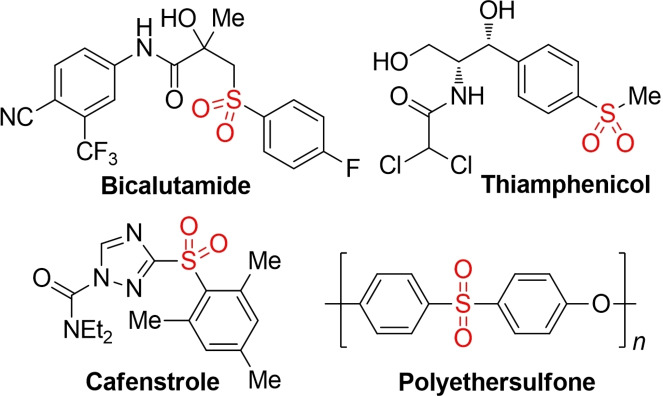
Structure of selected sulfones from different fields of application.

Among the different classes of organosulfur compounds, sulfones, that is, molecules containing a sulfonyl (−SO_2_−) functional group attached to two carbon substituents, are of particular importance.^[1]^ Sulfones are highly versatile building blocks for organic synthesis and can be utilized in a great variety of different transformations.^[2]^ Due to their ability to take part in various apparently different chemical processes, sulfones have been named “chemical chameleons”^[3]^ or “pluripotent”.^[4]^ Indeed, the reactivity of sulfones can be modulated from an electrophilic to a nucleophilic or even a radical character by adjustment of the reaction conditions.^[5]^ Furthermore, sulfones display a set of distinct structural and electronical properties, which in turn have led to a plethora of different applications in various fields ranging from agrochemicals and pharmaceuticals to functional materials.^[6]^ Selected examples include the anti‐cancer drug bicalutamide,^[7a]^ the antibiotic thiamphenicol,^[7b]^ the herbicide cafenstrole,^[7c]^ or polyethersulfone (PES), a high‐performance polymer^[7d]^ (Figure 1).

Considering this unique combination of versatile synthetic utility with a vast area of potential applications, the synthesis of sulfones has attracted considerable attention over time.^[8]^ The four traditional and still most common approaches for the construction of sulfones are the oxidation of sulfides (or sulfoxides), Friedel‐Crafts‐type reactions with sulfonyl chlorides, the electrophilic trapping of sulfinic acid salts, and the addition of sulfonyl radicals to alkenes or alkynes. Apart from those four classical methods, new procedures based on the selective functionalization of C−H bonds^[9a]^ and the fixation of sulfur dioxide^[9b]^ have emerged as novel, enabling tools in the last years.

In this Review, we want to highlight recent developments in the field of sulfone synthesis placing a particular focus on the progress towards greener or more sustainable methods. Therefore, this article will mainly focus on novel or improved methodologies utilizing solvents recommended by the Chem 21 selection guide.^[10]^ In the same manner, we will mostly cover novel procedures based on base‐metal catalysts or metal‐free processes. Methods employing problematic/hazardous solvents or reaction catalyzed by precious metals will only be covered if the reaction itself can serve as entry for the development for more sustainable methods. Within this context, we will not provide an exhaustive review over the whole field. Rather, progress (as well as still existing limitations) in terms of sustainability will be discussed on selected examples.

## 2 Synthesis via the Oxidation of Sulfides

The oxidation of sulfides is one of the oldest but still widely employed methods for the synthesis of sulfones.^[11a]^ Moreover, sulfide oxidation is performed on an enormous annual scale in the oxidative desulfurization, a process for the removal of sulfur compounds from crude oil.^[11b]^ Therefore, new approaches to improve the overall efficiency and sustainability of sulfide oxidation are still receiving great attention.

**Scheme 1:**
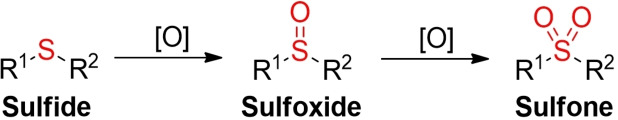
Oxidation of sulfides to sulfoxides and sulfones.

The oxidation of sulfides to sulfones is a two‐step process, which involves a sulfoxide as intermediate (Scheme 1). In general, the first oxidation of the sulfide to the sulfoxide is very facile and proceeds readily at low temperatures with only one equivalent of a suitable oxidant. The second step, the oxidation of the sulfoxide to the sulfone, usually requires more forcing conditions, such as elevated temperatures and excess of oxidant.

**Scheme 2:**
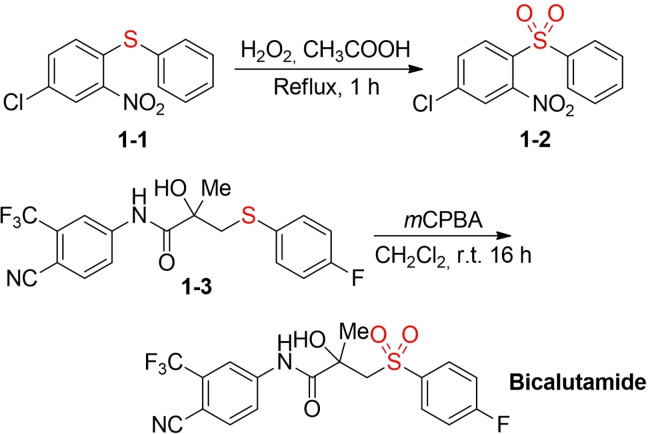
Selected examples for the synthesis of biologically active sulfones via sulfide oxidation.^[12]^

The most frequently employed oxidants for the synthesis of sulfones via sulfide oxidation are peracids and hydrogen peroxide in combination with acetic acid. In Scheme 2, two representative, arguably not sustainable examples from the recent literature are shown. Mayer and co‐workers prepared the sulfone **1**–**2**, an inhibitor of the mitotic motor protein Kif18 A, by refluxing the sulfide **1–1** in acetic acid in the presence of excess hydrogen peroxide.^[12a]^ The anticancer drug bicalutamide was synthesized for the first time by oxidation of the corresponding sulfide **1–3** with excess *meta*‐chloroperbenzoic acid (*m‐*CPBA).^[12b]^ Usually, these classical methods are associated with several disadvantages, such as great excess of the oxidant, highly acidic reaction conditions, and/or high temperatures.

Although a variety of other oxidants, such as nitric oxide, different metal oxides, hypervalent iodine reagents, halogenates or perhalogenates can be employed for the oxidation of sulfides to sulfones,^[13a]^ these procedures are all problematic in terms of toxic reagents, excess reagents, low chemoselectivity, or the use of hazardous organic solvents.^[13b]^

Therefore, a lot of effort has been devoted to the development of new catalysts for a more efficient and sustainable oxidation of sulfides to sulfones. In most studies H_2_O_2_, which can be considered as a “green” oxidant, is used.^[14]^ A variety of different homogenous and heterogenous catalyst systems have been studied for sulfide oxidation (Table 1).^[15]^ Most catalytic systems are based on inexpensive metals, such as titanium, iron, molybdenum, or tungsten (Table 1, entries 1–8). Metal‐free systems have been reported as well (Table 1, entries 9–12). Recent developments also include the implementation of a flow‐system based on a peroxometalate‐based polymer immobilized ionic liquid phase catalyst (Table 1, entry 7).

**Table 1:** Selected examples for sulfide oxidation with H_2_O_2_.

Entry	Type of sulfide			Catalyst	Oxidant	Solvent	Reaction conditions	Scope and yield	Ref.
	Ar−S−Ar	Ar−S‐alkyl	alkyl‐S‐alkyl						
1	yes	yes	yes	NbC (4 mol%)	H_2_O_2_ (30 % aq) (4.5 equiv.)	EtOH	60 °C	9 examples 90–99 %	[15a]
2	yes	yes	yes	MnSO_4_ ⋅ H_2_O (1 mol%)	H_2_O_2_ (30 % aq) (2.0–5.0 equiv.)	MeCN (but also DMF)	NaHCO_3_ buffer (0.2 m), RT, 0.25–24 h	15 examples 80–100 %	[15b]
3	yes	yes	no	TiO_2_‐400 (1.25 mol%)	H_2_O_2_ (35 % aq) (3.0 equiv.)	MeCN	40–80 °C, 3 h	5 examples 80–99 %	[15c]
4	yes	yes	yes	Fe_3_O_4_@BNPs@SiO_2_−SO_3_H (0.1 g mmol^−1^)	H_2_O_2_ (30 % aq) (4.0 equiv.)	EtOH	50 °C, 10–45 min	14 examples 90–98 %	[15d]
5	yes	yes	yes	[MoO_2_(O_2_)(L)_2_]^2−^‐MR (0.1 mol%)	H_2_O_2_ (50 % aq) (4.0 equiv.)	MeCN	RT: 100–410 min 78 °C: 35–170 min	12 examples >90 %, TON^[a]^≈970, TOF:^[b]^ 140–582 h^−1^ (RT), 345–1680 (78 °C)	[15e]
6	no	yes	yes	(C_19_H_42_N)_2_[MoO(O_2_)_2_(C_2_O_4_)] ⋅ H_2_O (2.5 mol%)	H_2_O_2_ (40 % aq) (3.0 equiv.)	H_2_O	RT, 1 min–8 h	18 examples 80–98 %	[15 f]
7	yes	yes	yes	[PO_4_{WO(O_2_)_2_}_4_]@PIILP (0.5 mol%)	H_2_O_2_ (35 % aq) (5.0 equiv.)	MeCN	45 °C, 15 min	7 examples 35–100 %, TOF: 280–800 h^‐1^	[15 g]
8	no	yes	yes	PDDA‐SiV_2_W_10_*^[c]^ (25 mg mmol^−1^)	H_2_O_2_ (30 % aq) (5.0 equiv.)	H_2_O	25 °C, 2–8 h	7 examples 99 %	[15 h]
9	yes	yes	yes	Amberlyst 15 56 mg mmol^−1^ sulfide	H_2_O_2_ (50 % aq) (3.5 equiv.)	AcOH	50 °C, 40–90 min	6 examples >99 %	[15i]
10	yes	yes	yes	BNPs−SiO_2_@(CH_2_)_3_NHSO_3_H (70 mol%)	H_2_O_2_ (30 % aq) (2.0 equiv.)	EtOH	50 °C, 10–45 min	18 examples 94–98 %, TON≈13–14	[15j]
11	yes	yes	yes	Borax (10 mol%)	H_2_O_2_ (35 % aq) (3.0 equiv.)	MeOH	pH=10–11, RT, 2.5–24 h	13 examples 10–95 %	[15k]
12	yes	yes	yes	PhenHTB (20–30 mol%)	H_2_O_2_ (30 % aq) (2.0 equiv.)	MeCN−H_2_O	RT, 25–95 min	10 examples 70–88 %	[15 l]

[a] TON=turnover number. [b] TOF=turnover frequency. [c] PDDA=poly(diallyldimethylammonium chloride).

The utilization of oxygen as terminal oxidant offers another attractive approach for a sustainable sulfide oxidation (Table 2).^[16]^ Different systems, both utilizing a metal catalyst or under metal‐free conditions, have been described (Table 2, entries 1–5). Interestingly, in most cases the formation of a reactive peroxide as active oxidant is postulated. In general, the oxidation with O_2_ seems to require higher temperatures than similar procedures using H_2_O_2_. Unfortunately, most reports with O_2_ as oxidant employ at least problematic organic solvents. For illustration, some selected examples are shown in Table 2, including two with acetonitrile, a solvent which is considered as nonproblematic by some solvent selection guides (entries 2 and 3).^[17]^

**Table 2:** Sulfide oxidation with O_2_.

Entry	Type of sulfide			Catalyst or promoter	Oxidant	Solvent	Reaction conditions	Scope and yield	Ref.
	Ar−S‐Ar	Ar−S‐alkyl	alkyl‐S‐alkyl						
1	yes	yes	no	[C_8_H_17_N(CH_3_)_3_]_3_HIV_9_O_28_ (40 mg per 0.3 mmol sulfide)	O_2_ (1 atm)	Decalin	90 °C, 4–12 h	4 examples 63–100 %	[11b]
2	yes	yes	no	Co‐SiO_2_@Ti−Si (50 mg per mmol sulfide) methylene hydrocarbon	O_2_ (1.0 MPa)	MeCN	120 °C, 4 h	6 examples 14–99 %	[16a]
3	no	yes	yes	2‐methyl‐1‐propanal (5.0 equiv.)	O_2_ (1 atm)	MeCN	70 °C, 3–6 h	7 examples 51–78 %	[16b]
4	yes	yes	no	–	O_2_ (1 atm)	BBE^[a]^	100 °C, 20 h	38 examples 63–95 %	[16c]
5	yes	yes	no	–	O_2_ (1 atm)	DPDME^[b]^	100 °C, 12–30 h	20 examples 48–98 %	[16d]

[a] BBE=bis(2‐butoxyethyl)ether. [b] DPDME=dipropylene glycol dimethyl ether.

As mentioned, most of these oxidation reactions are performed in classical, often highly flammable or toxic solvents. Therefore, considerable efforts have been devoted to the replacement of classical organic solvents with more sustainable alternatives.^[18]^ Several groups have shown that ionic liquids offer some advantages in the oxidation of sulfides, including a facile recycling of the ionic‐liquid catalyst system (Table 3, entries 1–4). Supercritical carbon dioxide has been successfully utilized as reaction medium for sulfide oxidation as well (Table 3, entry 5). Li and co‐workers demonstrated that sulfides can be oxidized to sulfones in water using oxone as the terminal oxidant (Table 3, entry 6).^[18f]^ Interestingly, sulfoxides are obtained as product using EtOH as solvent. Shankarling and co‐workers observed a similar reactivity (Table 3, entry 7). Rosati and co‐workers demonstrated a solvent‐free oxidation of sulfides to sulfones by mechanical milling with oxone (Table 3, entry 8). Other oxidants, such as sodium perborate (SPB) or *n*Bu_4_NHSO_5_ (TBAOX) also proved to be efficient in aqueous solutions (Table 3, entries 9 and 10).

**Table 3:** Sulfide oxidation in uncommon media or solvent‐free systems.

Entry	Type of sulfide			Catalyst	Oxidant	Solvent	Reaction conditions	Scope and yield	Ref.
	Ar−S‐Ar	Ar−S‐alkyl	alkyl‐S‐alkyl						
1	yes	yes	yes	V_2_O_5_ (20 mol%)	H_2_O_2_ (30 % aq) (2.5 equiv.)	[C_12_mim][HSO_4_]	RT–45 °C, 4–8 h	10 examples 82–95 %	[18a]
2	yes	yes	yes	–	[pmim]IO_4_ (4 equiv.)	[pmim]IO_4_	50 °C, 4–7 h	25 examples 74–92 %	[18b]
3	yes	yes	yes	–	NaBrO_3_ (3.0 equiv.)	[bmim]HSO_4_/H_2_O (3 : 1, *v*/*v*)	80 °C, 20–55 min	12 examples 83–92 %	[18c]
4	yes	yes	yes	[C_4_mim][ReO_4_] (5 mol%)	H_2_O_2_ (35 % aq) (4.0 equiv.)	[C_4_mim][BF_4_]	60 °C, 1–8 h	15 examples 56–97 %	[18d]
5	yes	yes	yes	–	[SiO_2_]‐CH_2_CH_2_CO_3_H (2.0 equiv.)	*sc*CO_2_ (250 bar)	flow condition: 0.10–0.12 mL *sc*CO_2_ min^−1^, 40 °C, 3 h	7 examples 72–99 %	[18e]
6	yes	yes	yes	–	oxone (1.5 equiv.)	H_2_O	60 °C, 12 h	10 examples 88–97 %	[18f]
7	yes	yes	yes	–	nonanebis(peroxoic acid) (1.5 equiv.)	H_2_O	50–55 °C, 25 min	9 examples 80–96 %	[18g]
8	yes	yes	yes	–	oxone (1.6 equiv.)	mechanical milling	90 min	6 examples >97 %	[18h]
9	no	yes	yes	–	sodium perborate (3.0 equiv.)	H_2_O	MW irradiation, 90 °C, 45 min	4 examples 39–99 %	[18i]
10	yes	yes	yes	CuPcS@ASMNP (0.5 mol%)	*n*Bu_4_NHSO_5_ (3.0 equiv.)	H_2_O	RT, 60 min	8 examples 70–97 %	[18j]

**Scheme 3:**
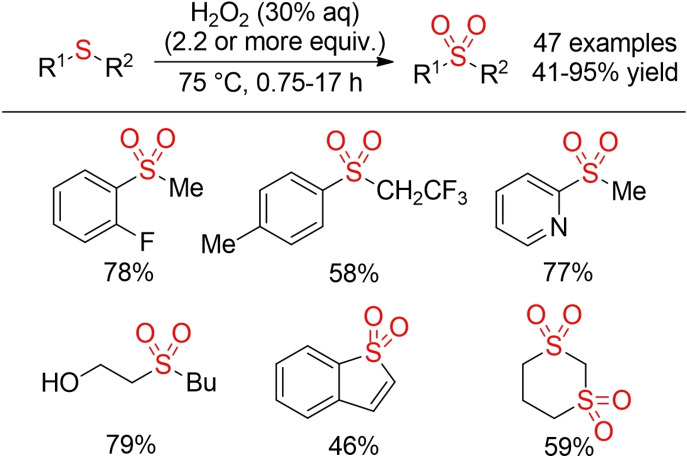
Oxidation of sulfides with H_2_O_2_ under catalyst‐ and solvent‐free conditions.^[19]^

From an environmental point of view, the method reported by Jereb in 2012 might offer the so far most sustainable approach for the oxidation of sulfides to sulfones (Scheme 3). Treatment of sulfides with a small excess of 30 % aq. H_2_O_2_ at 75 °C in the absence of any catalyst or additional solvent provides a variety of sulfones in good yields.^[19]^

In summary, the “green” oxidation of sulfides to sulfones has seen tremendous progress over the years and a variety of different environmentally benign procedures are available. Some of these transformations fulfil already most criteria for a truly green synthesis. One has to mention that in a lot of the discussed reports, only reactions with selected model compounds have been investigated. Of course, the general applicability of these procedures has yet to be demonstrated in laboratory settings and on an industrial scale. In addition, the use of highly active oxidizing species, such as O_2_ or H_2_O_2_, is always connected with safety issues. Therefore, there will be a continued interest in developing novel methods utilizing energy from renewable sources as driving force and implementing safer oxidation procedures.

## 3 Synthesis from Sulfonyl Chlorides and Other Sulfonic Acid Derivatives

**Scheme 4:**
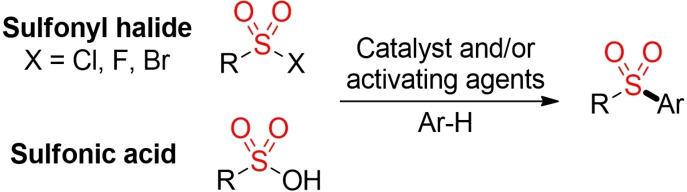
Friedel‐Crafts‐type sulfonylation of (hetero)arenes.

Aryl and heteroaryl sulfones can be prepared via the electrophilic aromatic substitution of the corresponding (hetero)arene with a suitable electrophilic sulfonylating agent in the presence of a Lewis or Brønsted acid (Scheme 4).[^1^, ^2^] Usually, sulfonyl chlorides are used as electrophilic reagents, but reactions with other sulfonyl halides or sulfonic acids have been reported as well. In general, these reactions are performed in the presence of stoichiometric amounts of a Lewis or Brønsted acid, such as AlCl_3_, FeCl_3_, or phosphoric acid.^[20]^ As in the case of any other electrophilic aromatic substitution, electron‐donating or ‐withdrawing groups have a strong influence on the reactivity of the (hetero)arene and the regioselectivity of the reaction.^[21]^

All these methods suffer from the typical drawbacks of Friedel−Crafts‐type transformations, such as harsh reaction conditions, high temperatures, and often a low regioselectivity. The use of stoichiometric amounts of an acid leads to the generation of substantial quantities of waste and can cause safety issues during the reaction and the workup process. Therefore, considerable efforts have been devoted to the development of more efficient and sustainable methods (Table 4).^[22]^ Special emphasis has been placed on the identification of metals salts, which can be employed in low amounts, such as Bi^3+^‐salts (entries Table 4, 1–3). Other groups have focused on the development of solid‐supported, reusable catalysts (Table 4, entries 4 and 5), ionic‐liquid‐based systems (Table 4, entries 6–8), or under microwave irradiation (Table 4, entries 9 and 10). However, careful evaluation of all these improvements reveals that every method still displays some disadvantages in terms of its sustainability. Either high temperatures or high catalyst loadings are needed, or toxic organic solvents are utilized. Contrary to the sulfide oxidation, highly sustainable procedures for a Friedel–Crafts‐type sulfonylation are still missing.

**Table 4:** Electrophilic aromatic sulfonylation with sulfonyl chloride or sulfonic anhydride.

Entry	Catalyst	Sulfonylating agent	Solvent	Reaction conditions	Scope and yield	Ref.
1	Bi(OTf)_3_ (1–10 mol%)	RSO_2_Cl, RSO_2_OSO_2_R ArH/RSO_2_X=2 : 1 (mol)	–	90–120 °C, 1–7 h	16 examples 35–90 %	[22a]
2	Cu(OTf)_2_ (10 mol%) or Sn(OTf)_2_ (5 mol%)	ArSO_2_Cl ArH/ArSO_2_Cl=3 : 1 (mol)	–	120 °C, 8–12 h	26 examples 30–99 %	[22b]
3	BiCl_3_/TfOH (10 mol%) or SbCl_3_/TfOH (10 mol%)	MeSO_2_Cl ArH/MeSO_2_Cl=3 : 1 (mol)	–	105–130 °C, 0.7–7 days	13 examples 19–97 %	[22c]
4	Ps‐AlCl_3_ (15 mol%) SiO_2_‐AlCl_3_ (10 mol%)	TsCl, PhSO_2_Cl ArH/RSO_2_Cl=2 : 1 (mol)	–	85 °C, 1–2.3 h	18 examples 87–95 %	[22d]
5	zeolite Hβ (0.6 g) or Naβ (1.0 g)	(MeSO_2_)_2_O (7.0 mmol)	ArH (15 mL)	reflux, 18 h	12 examples 12–78 %	[22e]
6	AlCl_3_ (mole fraction N=0.67)	TsCl ArH/TsCl=1.1 : 1 (mol)	[bmim]Cl‐AlCl_3_ (1.2 equiv.)	30–50 °C, 3–5 h	6 examples 83–92 %	[22 f]
7	FeCl_3_ (N=0.5)	TsCl, PhSO_2_Cl ArH/RSO_2_Cl=1 : 1 (mol)	[BTBA]Cl–FeCl_3_ (1.0 equiv.)	60 °C, 1–5 min	20 examples 90–97 %	[22 g]
8	[bmim]Cl⋅FeCl_3_ (5–10 mol%, N=0.6)	TsCl, PhSO_2_Cl ArH/RSO_2_Cl=2 : 1 (mol)	–	80–135 °C, 0.5–12 h	11 examples 58–93 %	[22 h]
9	[bmim]Cl⋅FeCl_3_ (5–10 mol%, N=0.6)	TsCl ArH/TsCl=2 : 1 (mol)	–	microwave, 80–165 °C, 3–30 min	9 examples 29–91 %	[22 h]
10	FeCl_3_ (5–10 mol%)	ArSO_2_Cl, Ts_2_O ArH/RSO_2_Cl=2 : 1 (mol)	–	microwave, 110–254 °C, 45 s–20 min	19 examples 50–95 %	[22i]

Apart from that, electrophilic aromatic substitutions with sulfonyl halides are always limited by their inherent regioselectivity and will lead to the generation of one equivalent of a hydrogen halide as by‐product.

In this context, sulfonic acids or sulfonate salts can serve as (at least in principle) more sustainable electrophilic agents, furnishing the desired sulfone and water as the only by‐product However, older methods are mostly based on the in‐situ activation of the sulfonic acid with stoichiometric amounts of an external activating agent, such as Tf_2_O, which again leads to generation of problematic waste products.^[23]^ The identification of solid‐supported acid catalysts, such as Nafion‐H, has led towards more sustainable methods for the direct sulfonylation with sulfonic acids in the absence of stoichiometric activating agents (Table 5).^[24]^

**Table 5:** Electrophilic aromatic sulfonylation with sulfonic acids.

Entry	Catalyst or promoter	Sulfonylating agent	Solvent	Conditions	Scope and yield	Ref.
1	P_2_O_5_/SiO_2_ (*w*/*w* 75 %, 1.2 g)	TsOH, PhSO_3_H, MsOH (3.1 mmol)	ArH (5 mL)	reflux, 30–105 min	15 examples 50–90 %	[24a]
2	Nafion‐H (400 mg, 50 wt%)	TsOH, PhSO_3_H, MsOH (5.0 mmol)	ArH (40 mL)	reflux, 8–20 h, Dean–Stark trap	11 examples 30–82 %	[24b]
3	Fe^3+^‐montmorillonite (0.2 g)	ArSO_3_H, MsOH, Ms_2_O, Ts_2_O (3.0 mmol)	ArH (5 mL)	reflux, 6–24 h	18 examples 23–94 %	[24c]
4	SiO_2_−AlCl_3_ (10 mol%)	TsOH, PhSO_3_H ArH/ ArSO_3_H=4 : 3 (mol)	–	80 °C, 1.3–2.7 h	22 examples 85–94 %	[24d]

In general, the direct electrophilic aromatic sulfonylation is well suited for electron‐rich arenes and heteroarenes. Unfortunately, reactions with electron‐deficient substrates are rarely documented in the literature. Most of the above‐mentioned reports do not provide any details on reactions with electron‐poor (hetero)aromatics. In addition, the electrophilic aromatic sulfonylation always leads to the formation of one (or more) regioisomers, solely governed by the inherent reactivity of the (hetero)aromatic starting material.

In the last twenty years, the regioselective functionalization of distinct C−H bonds exploiting a combination of transition metal catalysis and directing groups has led to tremendous progress in organic synthesis.^[25]^ With some delay, the field of transition‐metal catalyzed C−H functionalization has been expanded towards the direct formation of C−S bonds, including direct sulfonylation reactions with sulfonyl chlorides.[^9a^, ^26^] Since the initial report by Dong and co‐workers,^[27a]^ the transition‐metal catalyzed functionalization of C−H bonds with sulfonyl chlorides has become an attractive and (in terms of regioselectivity) complementary approach for the sustainable synthesis of sulfones.

**Scheme 5:**
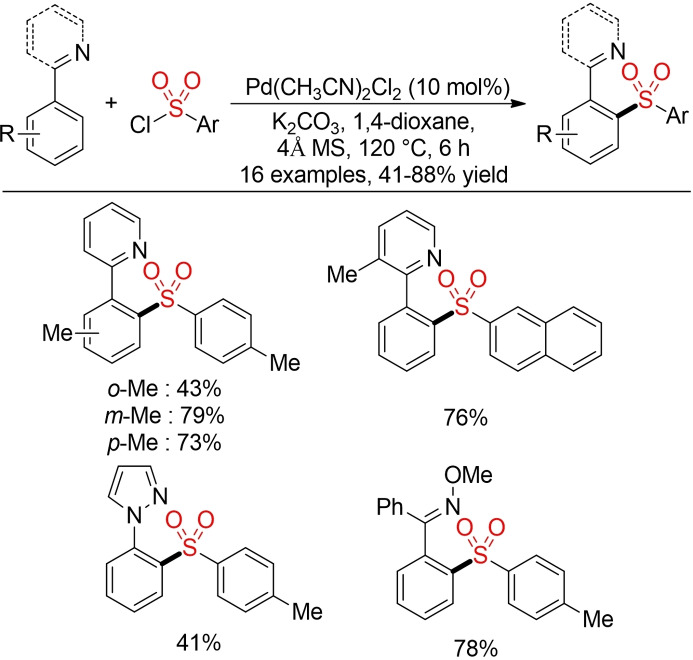
Palladium‐catalyzed C−H sulfonylation of phenylpyridines with arene sulfonyl chlorides.^[27]^

In 2009 Dong and co‐workers reported the first palladium‐catalyzed C−H sulfonylation of phenylpyridines with arene sulfonyl chlorides (Scheme 5).^[27]^

Since this pioneering report various groups have developed protocols for the direct sulfonylation of aromatic as well as vinylic C(sp^2^)−H bonds with arene sulfonyl chlorides utilizing different directing groups. However, many of these procedures use either rare transition‐metal catalysts (e. g., palladium or rhodium) or employ problematic organic solvents, just to mention 1,2‐dichloroethane (DCE) as still the most common solvent in C−H‐functionalization reactions.^[28]^ Although various groups have shown that base metals catalysts, such as Ni,^[29]^ Cu,^[30]^ or Ru,^[31]^ can be utilized for the direct sulfonylation of C−H bonds, the use of hazardous solvents is still preeminent. Overall, the direct functionalization of C−H bonds holds tremendous potential for the development of more sustainable synthetic methods. However, one has to emphasize that all until now described procedures are far from perfect. Almost all reports focus primarily on the discovery of new reactivity profiles and neglect sustainability aspects (in particular the choice of solvent). As a consequence, most methods still rely on (highly) hazardous organic solvents, such as DCE or dioxane, which cannot be recommended from an environmental perspective. Furthermore, the use of stoichiometric amounts of additives and/or bases leads to additional byproducts. In order to render these metal‐catalyzed C−H sulfonylation reactions truly sustainable, considerable efforts are still needed. In addition, most reports are limited to the utilization of arene sulfonyl chlorides and so far, no metal‐catalyzed direct sulfonylation of C(sp^3^)−H bonds with sulfonyl chlorides has been described. Although the field of metal‐catalyzed C−H functionalization holds great potential for the future, the transformation of these initial discoveries into truly sustainable methods for the synthesis of sulfones is still needed.

## 4 Synthesis from Sulfinic Acid Salts (and Derivatives)

Sulfinic acid salts (or sulfinates) are versatile building blocks for the synthesis of sulfones.^[32]^ These ambident nucleophiles react with a variety of electrophiles, predominantly at the sulfur center.

**Scheme 6:**
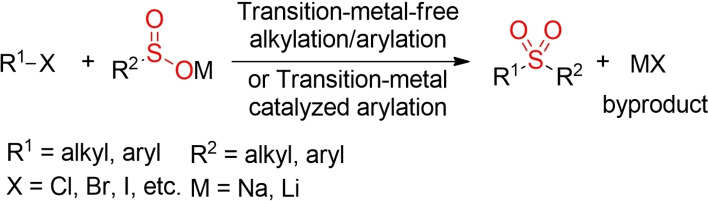
Alkylation/arylation of sulfinic acid salts with alkyl/aryl halides.

The alkylation of sulfinic acid salts with different alkyl halides has been utilized for the synthesis alkyl sulfones for more than 75 years.[^2^, ^33^] In a similar manner, sulfinates can be transformed into aryl sulfones using either transition‐metal catalyzed coupling reactions^[34]^ with aryl halides or direct, metal‐free arylation reactions^[35]^ with activated (hetero)aryl halides (Scheme 6). However, all these reactions lead to the generation of at least one equivalent of metal salt byproduct. In recent years, various groups have explored alternative procedures for a more sustainable alkylation of sulfinate salts.

**Scheme 7:**
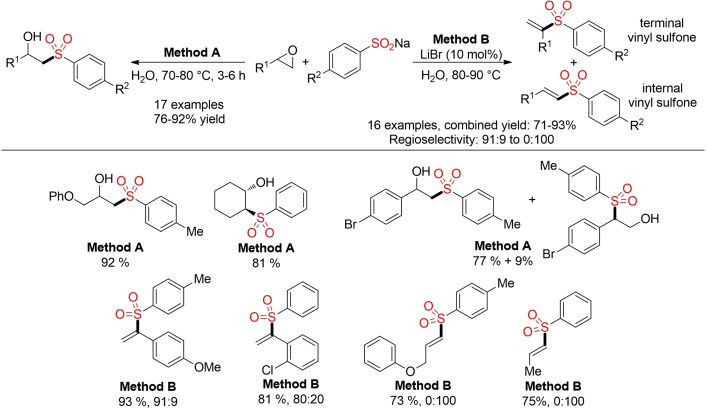
Synthesis of sulfones via direct opening of epoxides with sodium sulfinates.^[36]^

The direct alkylation of sulfinates with epoxides provides a more atom‐economic approach for the preparation of sulfones. The direct opening of epoxides with sodium sulfinates on water as solvent leads to the corresponding β‐hydroxy sulfones in good yields.^[36a]^ In contrast, the same reaction in the presence of 10 mol% LiBr affords the corresponding vinyl sulfones, presumably via an epoxide opening–elimination sequence (Scheme 7).^[36b]^ Both reports showcase the potential of epoxides as more atom‐economic building blocks for the synthesis of sulfones.

**Scheme 8:**
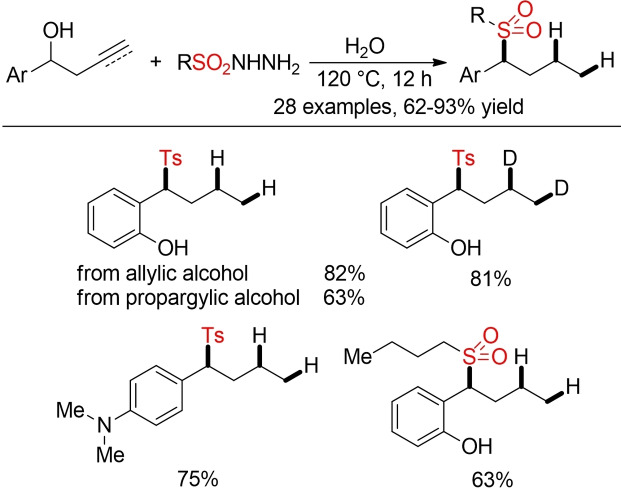
Sulfonylation of homoallylic or homopropargylic alcohols with sulfonyl hydrazides.^[37]^

Zhang and co‐workers described an intriguing sulfonylation of homoallylic or homopropargylic alcohols with sulfonyl hydrazides (Scheme 8).^[37]^ The sulfonyl hydrazide serves as a sulfonyl source and as reductant for the concomitant hydrogenation of the alkene or alkyne. The reaction is performed in water as solvent in the absence of any catalyst or additive. By using D_2_O as solvent, a selective deuteration of the alkene can be accomplished.

**Scheme 9:**
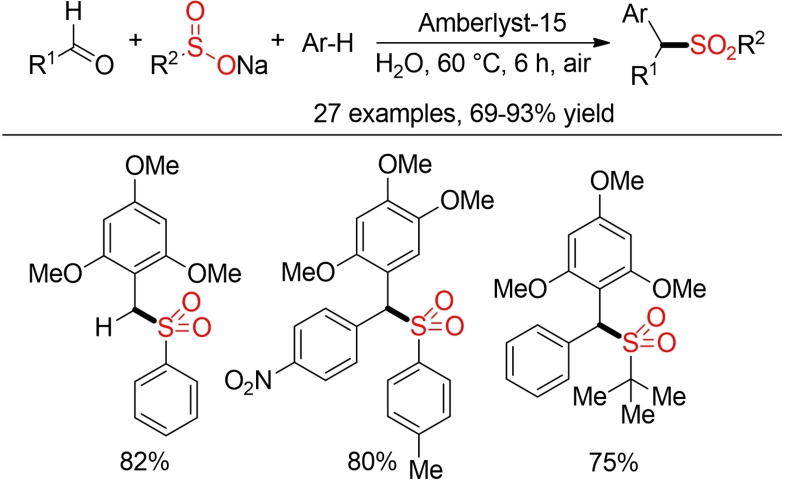
Amberlyst‐15‐catalyzed three‐component sulfonylation.^[38]^

Diarylmethyl sulfones can be accessed in a three‐component reaction starting from aryl aldehydes, sodium sulfinates, and electron‐rich (hetero)arenes. The reaction proceeds efficiently in water in the presence of a recyclable Amberlyst‐15 resin as catalyst (Scheme 9).^[38]^

**Scheme 10:**
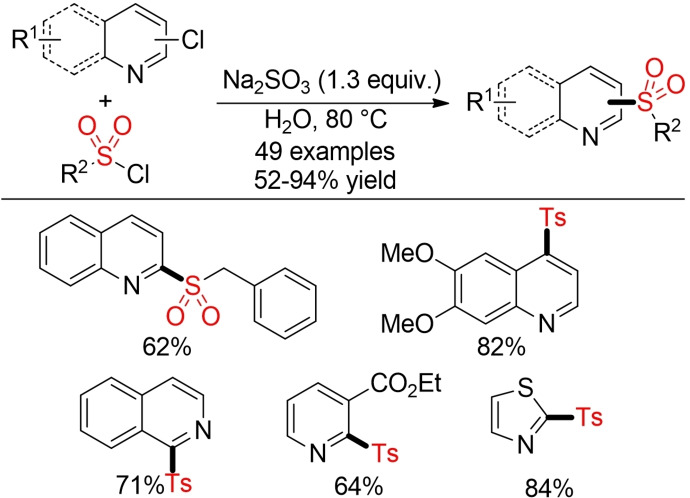
Synthesis of sulfonylated N‐heteroaromatics in water.^[39]^

He and co‐workers described a waste‐minimized one‐pot protocol for the synthesis of sulfonylated pyridines and quinolines (Scheme 10).^[39]^ In this method, the necessary sulfinic acid salts are generated in situ from the corresponding sulfonyl chlorides and Na_2_SO_3_. As sulfinates are mostly prepared from sulfonyl chlorides anyway, this process obviates a time‐ and resource‐consuming additional step. Moreover, the authors demonstrated that their process is amendable to scale‐up and the products can be isolated by a simple filtration procedure.

**Scheme 11:**
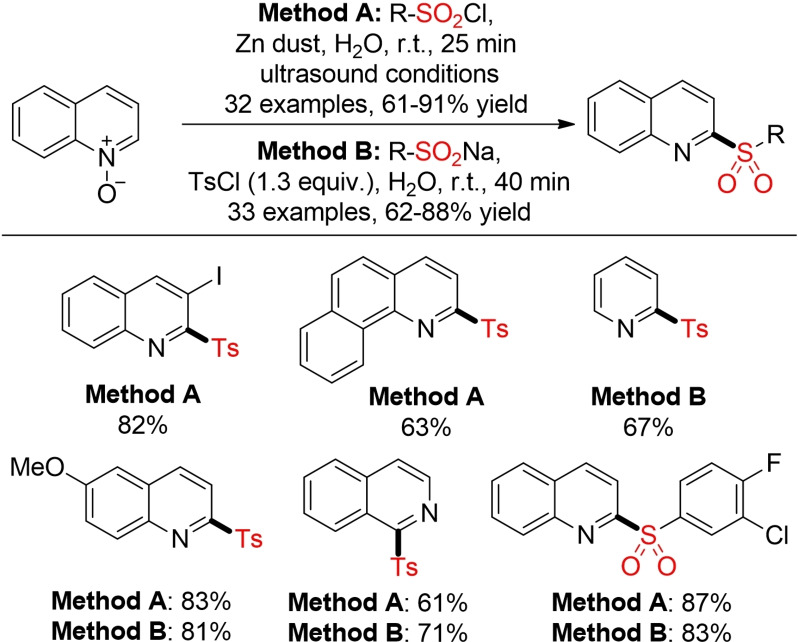
Synthesis of quinoline‐*N*‐oxides in water.^[40]^

A similar protocol was developed for the sulfonylation of quinoline‐*N*‐oxides. In this case, zinc dust serves both as reductant for the sulfonyl chloride and the *N*‐oxide. Later on, He and co‐workers described an analogous method for the direct sulfonlyation with sulfinic acid salts using tosyl chloride as promoter. (Scheme 11).^[40]^

**Scheme 12:**
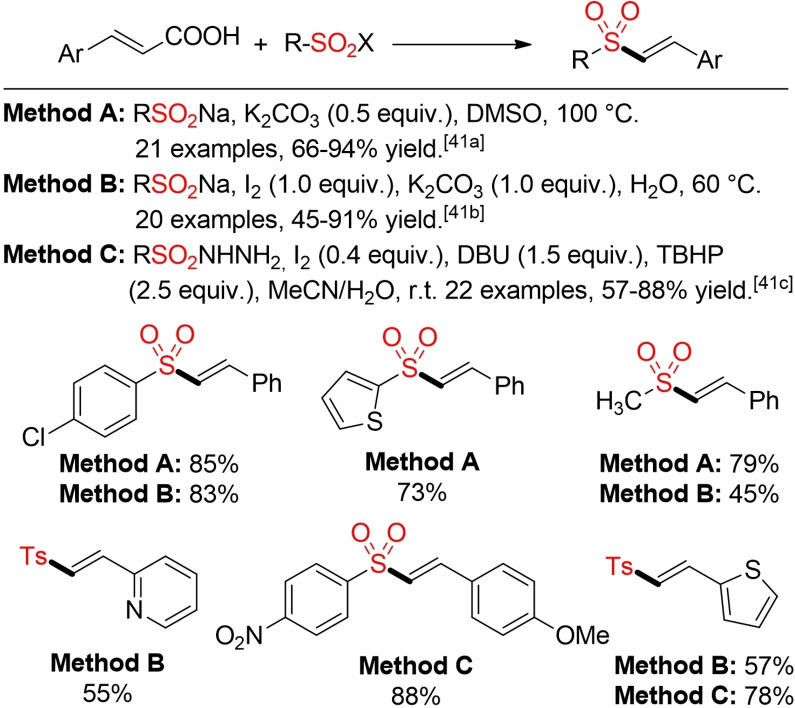
Decarboxylative sulfonylation of cinnamic acids.^[41]^

Jiang and co‐workers developed a novel approach for the decarboxylative sulfonylation of olefinic carboxylic acids (Scheme 12).^[41a]^ This method opens a new opportunity for a more sustainable construction of the sulfone scaffold with carbon dioxide as environmentally more benign byproduct. Indeed, two more examples for decraboxylative coupling of cinnamic acids with sodium sulfinates and sulfonyl hydrazides, both promoted by iodine, have been disclosed afterwards.[^41b^, ^41c^]

As already discussed, the development of novel methods for the synthesis of C−S bonds via the selective functionalization of C−H bonds has become an attractive approach for the development of more sustainable methods for the synthesis of sulfones.[^9a^, ^26^] In parallel to the above‐mentioned procedures employing sulfonyl chlorides, various groups have focused on the oxidative coupling^[42]^ of sulfinic acid salts with different C−H bonds.

**Scheme 13:**
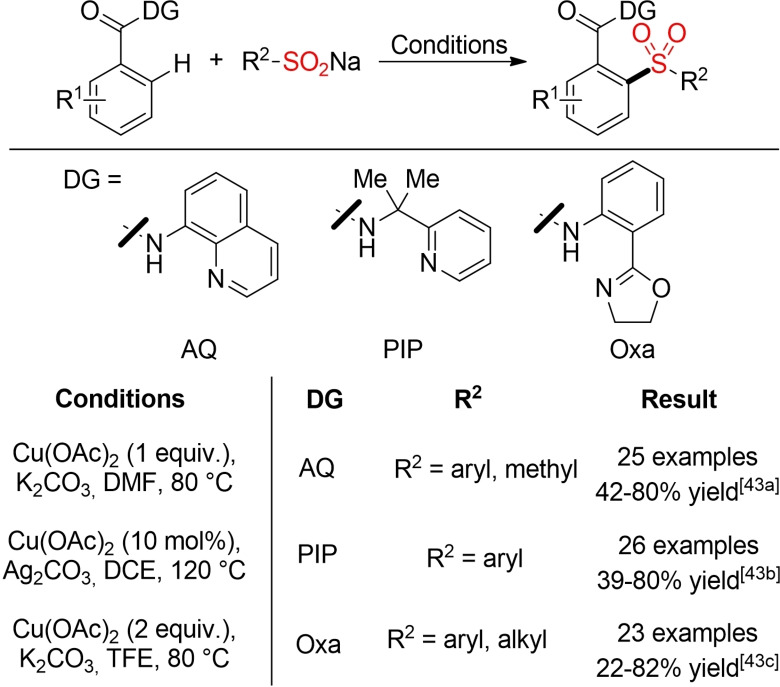
Copper‐catalyzed/mediated *ortho*‐sulfonylation of benzamide derivatives.^[43]^

In three initial reports, Tan and co‐workers, Rao and Shi, and Manolikakes and co‐workers describe a copper‐catalyzed or ‐mediated oxidative *ortho*‐sulfonylation of benzamide derivatives bearing different directing groups (Scheme 13).^[43]^ Although, these methods provide a complementary entry into the direct sulfonylation of C−H bonds, the sustainability profile of all three reactions is highly unfavorable [stoichiometric amounts of a copper(II) salt, additional base additives, hazardous solvents].

**Scheme 14:**
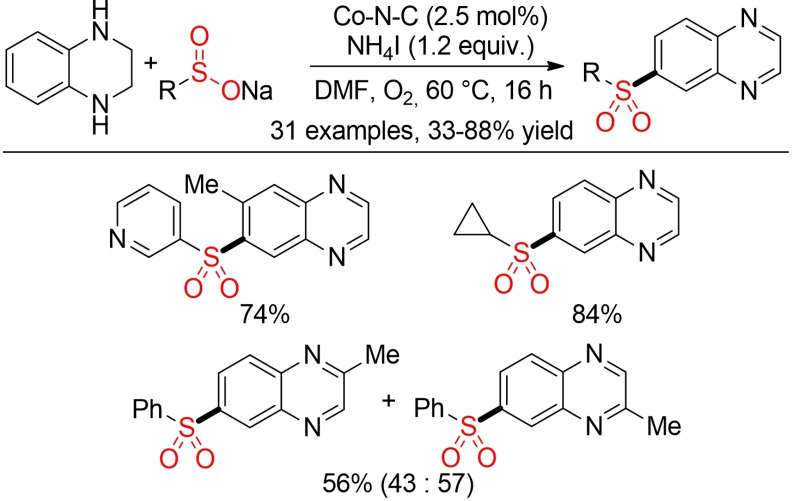
Cobalt‐catalyzed selective oxidative sulfonylation of tetrahydroquinoxalines.^[45]^

Based on these initial reports, various groups reported novel methods for metal‐catalyzed oxidative coupling of sulfinic acid salts with different C−H‐bonds.^[44]^ In almost all cases, the focus is solely on establishing new reactions and reactivity profiles. In addition, the requirement for a tailored directing group necessitates the additional synthetic steps for the introduction and removal of these groups. Sustainability aspects still have to be addressed in the future. Herein, we just want to discuss one report from Zhang and co‐workers for the synthesis of sulfonylated quinoxalines. This method utilizes a heterogeneous and recyclable metal–organic framework (MOF)‐based Co‐catalyst together with O_2_ as terminal oxidant both for the coupling itself and for a subsequent oxidation of the tetrahydroquinoxaline starting material, albeit in DMF as solvent (Scheme 14).^[45]^

An environmentally more benign (but arguably more restricted) approach is the metal‐free, direct oxidative sulfonylation of various substrates exploiting their inherent reactivity.

**Scheme 15:**
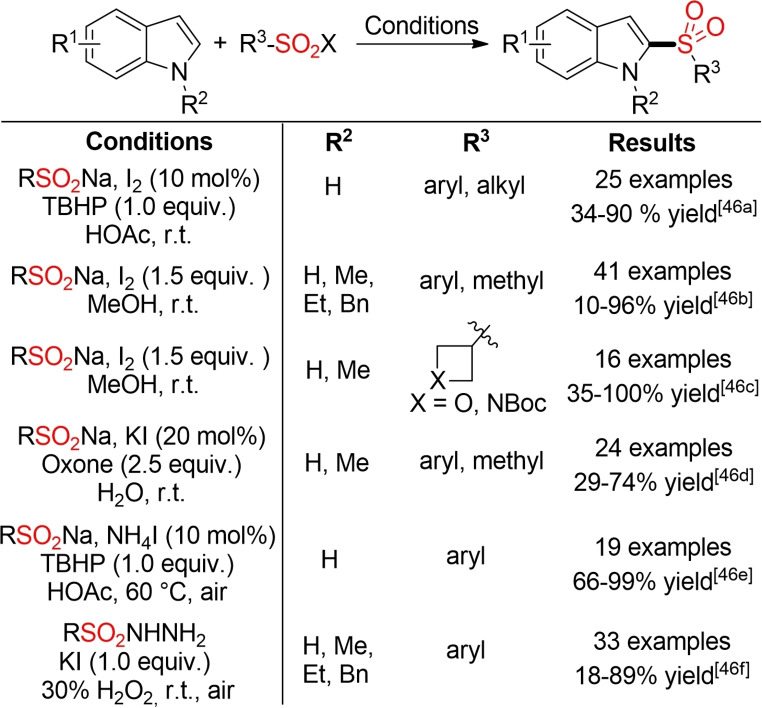
Iodine‐catalyzed/mediated C2‐selective oxidative sulfonylation of indoles.^[46]^

The C2‐selective oxidative sulfonylation of indoles with sulfinic acid salts or sulfonyl hydrazides reported by several groups represents a good example for this type of process (Scheme 15).^[46]^ Interestingly, all five reports employ iodine either as mediator (stoichiometric amounts of I_2_) or catalyst (in combination with a terminal oxidant) for the sulfonylation of the indole scaffold.

**Scheme 16:**
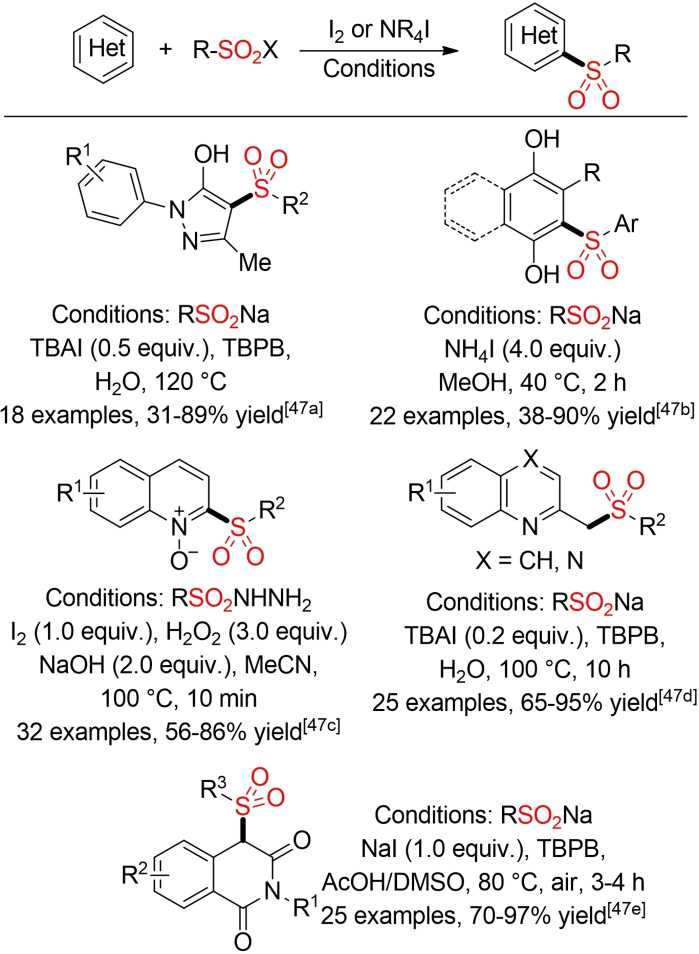
Iodine/Iodide‐promoted oxidative sulfonylation of (hetero)arenes.^[47]^

Iodine/Iodide can serve as efficient promoter for the direct oxidative C−H‐sulfonylation of other (hetero)aromatic scaffolds, such as pyrazolones, quinones, quinoline‐*N*‐oxides, or isoquinoline‐1,3(2*H*,4*H*)‐diones and for the synthesis of sulfomethyl azaarenes (Scheme 16).^[47]^

**Scheme 17:**
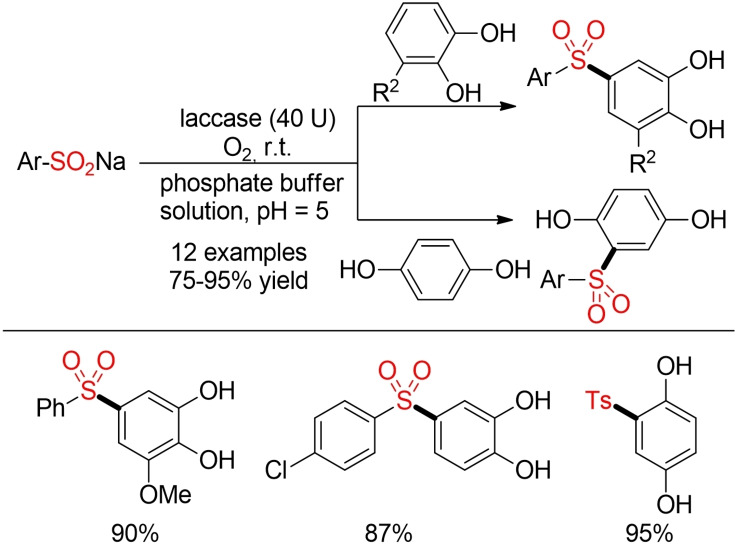
Laccase‐catalyzed aerobic oxidative coupling.^[48]^

The laccase‐catalyzed oxidative sulfonylation of catechols or dihydroquinones is a notable extension in the field of oxidative sulfinate coupling. It not only utilizes oxygen as terminal oxidant but also represents a rare example for a biochemical synthesis of sulfones (Scheme 17).^[48]^

**Scheme 18:**
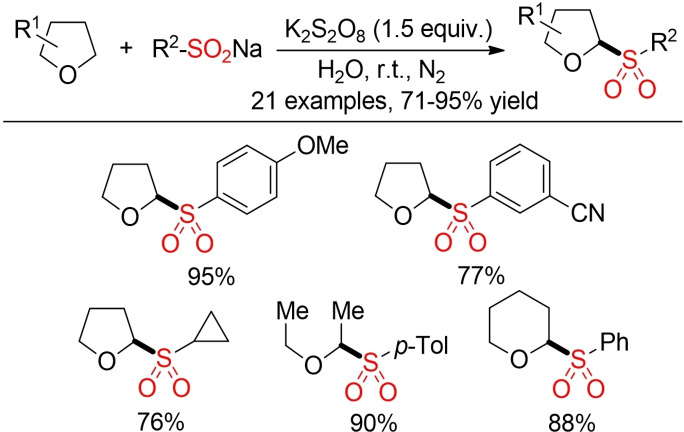
Sulfonylation of tetrahydrofurans with sulfinic acid salts.^[49]^

Examples for the direct oxidative coupling of C(sp^3^)−H bonds are rare. One example is depicted in Scheme 18. The direct C(sp^3^)−H sulfonylation of tetrahydrofurans could be achieved with K_2_S_2_O_8_ as oxidant in water as reaction medium.^[49]^

Although the synthesis of sulfones via sulfinic acid salts as building blocks has progressed considerably over the years, all methods display one inherent disadvantage with regard to their overall sustainability. The required sulfinate salts are usually prepared by the reduction of the corresponding sulfonyl chlorides, which in turn are prepared from other pre‐functionalized starting materials in one or more steps. With respect to this inherent limitation, it will be difficult to imagine highly sustainable methods for the synthesis of sulfones based on sulfinates. However, progress made in this area can still lead to the discovery of new reactivities and methods, which in turn can be further advanced towards a truly sustainable sulfone synthesis.

## 5 Synthesis via Radical Addition to Double and Triple Bonds

**Scheme 19:**
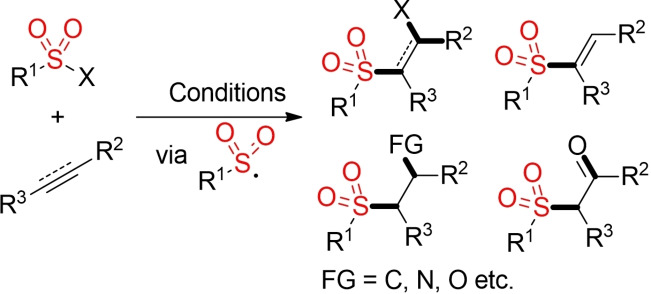
Addition of sulfonyl radicals to olefins and alkynes.

The addition of in‐situ generated sulfonyl radicals to olefins and alkynes represents another classical approach for the construction of sulfones.^[50]^ As for every addition reaction, these transformations feature a high atom‐economy (Scheme 19). Furthermore, these methods offer a versatile approach to various different sulfonyl group‐containing scaffolds. The required sulfonyl radicals can be generated from a variety of different precursors in a facile manner. Addition under oxidative conditions or with subsequent elimination of a leaving group leads to electron‐deficient alkenes or alkynes, which are useful building blocks for further synthetic manipulations. “Real” addition reactions provide β‐functionalized sulfones, either with incorporation of another functionality from the sulfonyl precursors or from an external reaction partner.

Since the initial reports on the addition of sulfonyl chlorides to alkenes,^[51]^ considerable improvements have been achieved. Herein we want to cover some more recent examples with particular significance for a greener sulfone synthesis. However, the most interesting developments in this field will be covered in the upcoming chapters dealing with novel photo‐ and electrochemical methodology developments.

**Scheme 20:**
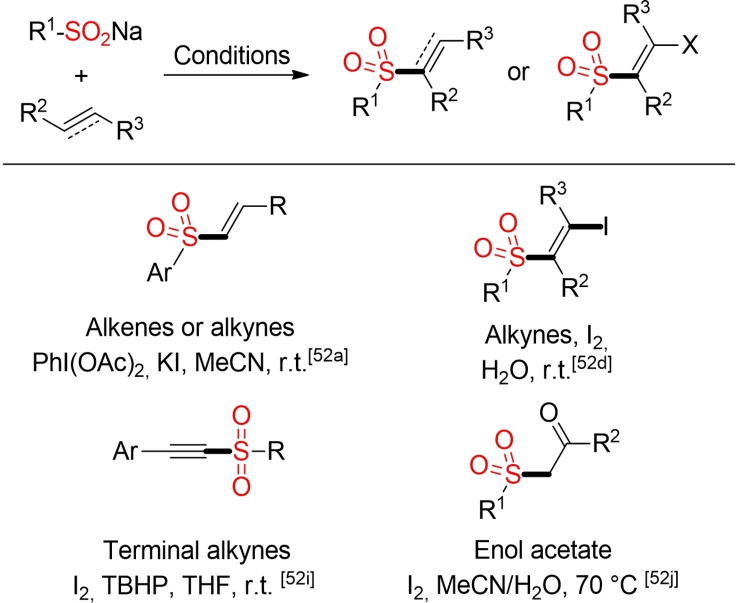
Selected metal‐free addition reactions of sulfinates to double and triple bonds.^[52]^

In the last ten years various groups reported different procedures for metal‐free addition reactions of sulfinic acids and their salts to double and triple bonds.^[52]^ In most cases, iodine serves as mediator or promoter for the addition reaction and the iodosulfonylation product is obtained. For the addition to alkenes, the formed β‐iodoalkyl sulfones are mostly fleeting intermediates towards the final vinyl sulfone products. Notably, the iodosulfonylation of alkynes can be even performed in water at ambient temperature (Scheme 20).

**Scheme 21:**
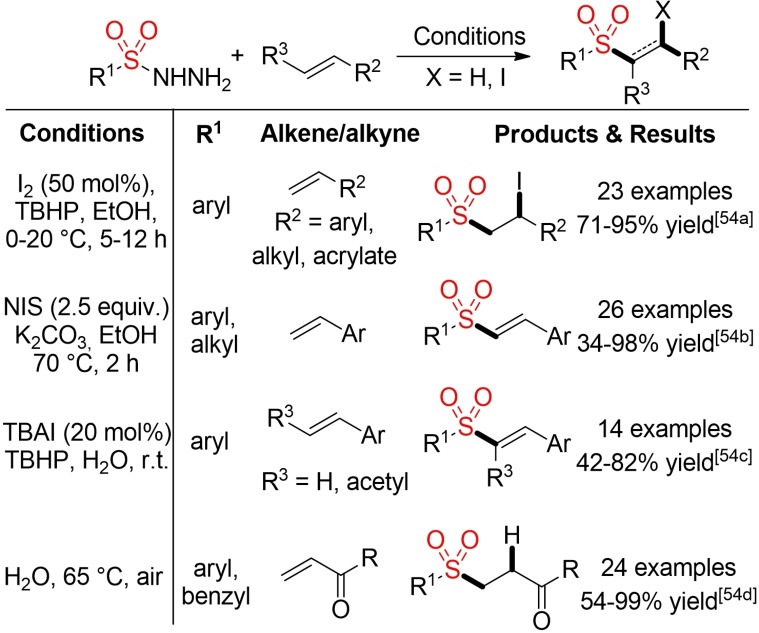
Iodine/iodide‐mediated sulfonylation of alkenes.^[54]^

Sulfonyl hydrazides provide another stable and easily accessible precursor for sulfonyl radicals.^[53]^ The required radicals can be generated via oxidative cleavage of the hydrazide employing different types of oxidants. Herein, only metal‐free, iodine/iodide‐mediated processes will be shown. It is worth mentioning that some of these processes proceed efficiently in water as solvent. As before, the outcome of these reactions (iodosulfonylation vs. formal hydrosulfonylation) is governed by the substrate and reaction conditions (Schemes 21, ^[54]^ and 22[^54c^, ^55^]).

**Scheme 22:**
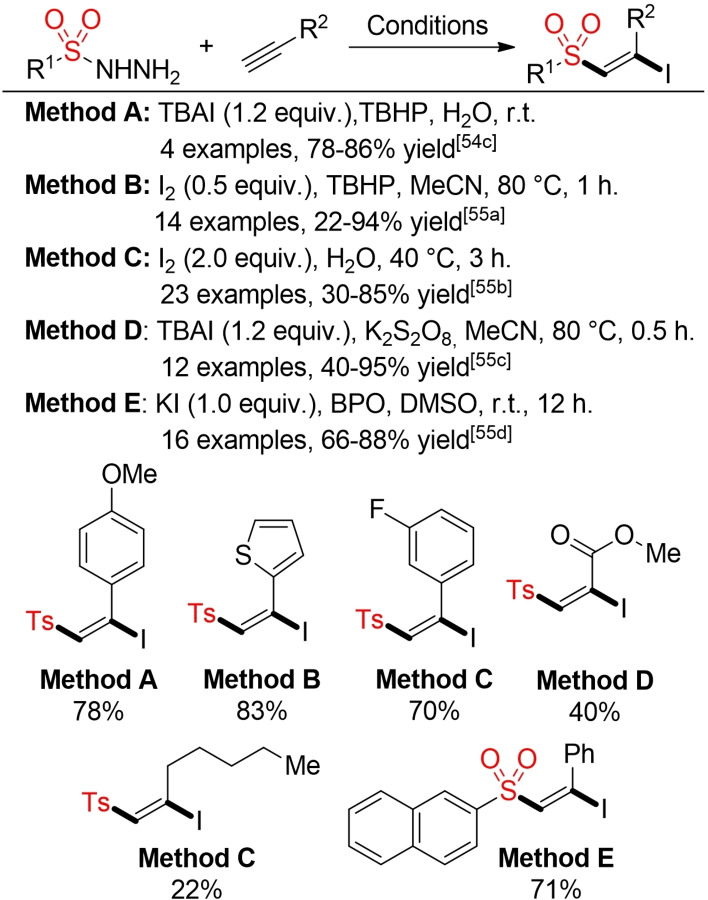
Iodine/iodide‐mediated sulfonylation of alkyenes.[^54c^, ^55^]

**Scheme 23:**
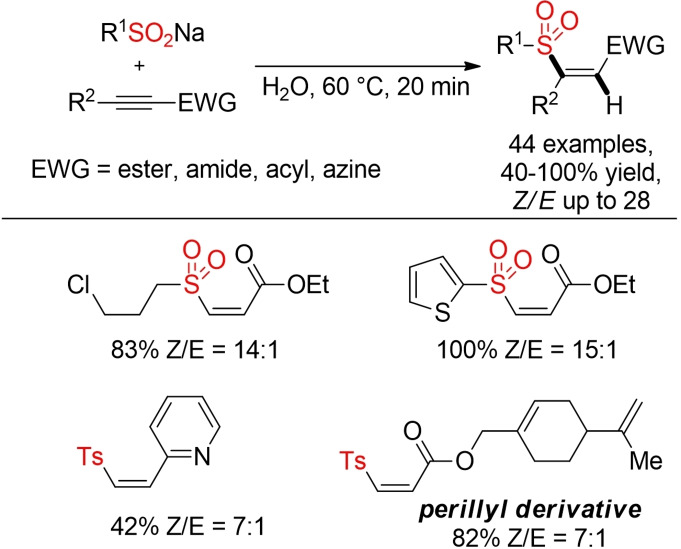
Sulfonylation of electron‐deficient alkynes in H_2_O.^[56]^

An interesting example for regioselective and stereoselective sulfonylation of electron‐deficient alkynes has been described by He and co‐workers (Scheme 23).^[56]^ This Michael‐type addition proceeds readily in water as solvent at 60 °C, furnishing the *Z*‐configured vinyl sulfones in high yields.

**Scheme 24:**
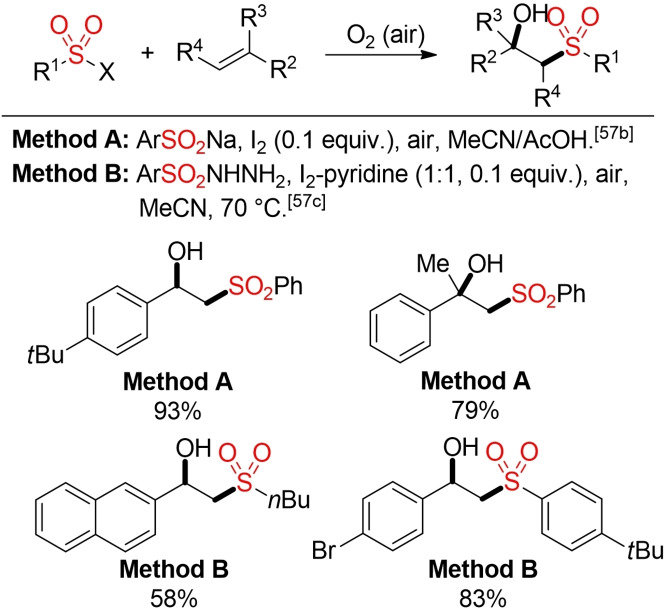
Aerobic oxysulfonylation of alkenes.[^57b^, ^57c^]

In the presence of oxygen as terminal trapping agent, an oxysulfonylation of alkenes can occur. Various protocols for the synthesis of β‐hydroxysulfones from alkenes, oxygen (or air), and different sulfur‐based building blocks, such as sulfinic acids or their salts or sulfonyl hydrazides, have been described in the last ten years.^[57]^ Notably, these transformations can be initiated by a variety of different metal‐free catalysts or additives, and in most cases the use of air instead of pure oxygen gas is sufficient. One should pay attention that in all cases potentially labile hydroperoxide species are formed as intermediates. Depending on the protocol, the hydroperoxides are directly reduced in the corresponding transformation or the use of an external, terminal reductant, such as PPh_3_, is necessary. The latter case not only leads to additional by‐products but also to an accumulation of reactive hydroperoxides, which can result in safety issues, especially for large‐scale reactions.^[57a]^ However, sulfinic acid salts can also serve as internal reductant, leading on the one hand to a direct reduction of the hydroperoxides. On the other hand, an additional equivalent of the sulfinate is consumed in a nonproductive pathway, that is, without incorporation into the final product (Scheme 24).[^57b^, ^57c^]

**Scheme 25:**
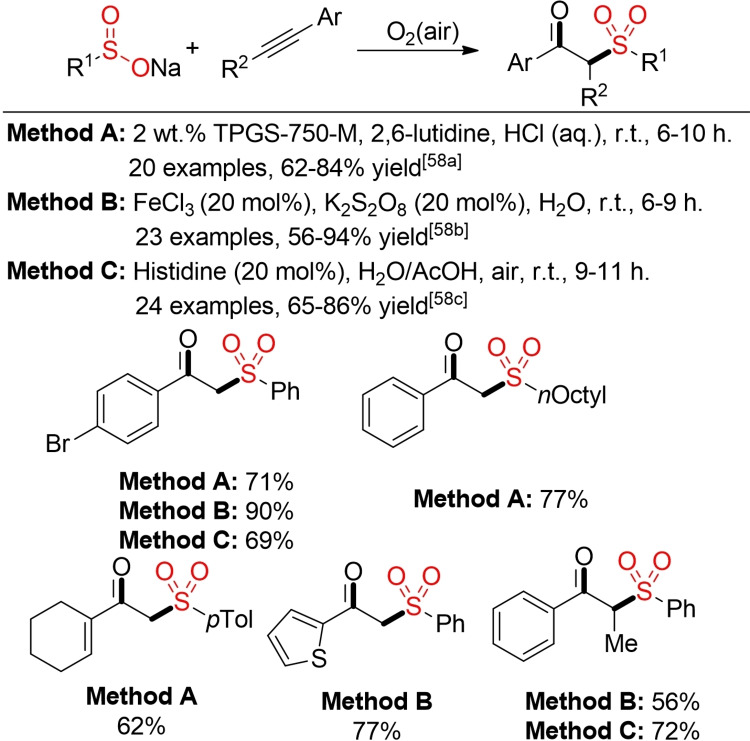
Synthesis of β‐keto sulfones.^[58]^

An analogous addition of sulfonyl radicals, generated from sulfinic acid salts, to alkynes in the presence of oxygen furnishes the corresponding β‐keto sulfones (Scheme 25).^[58]^

**Scheme 26:**
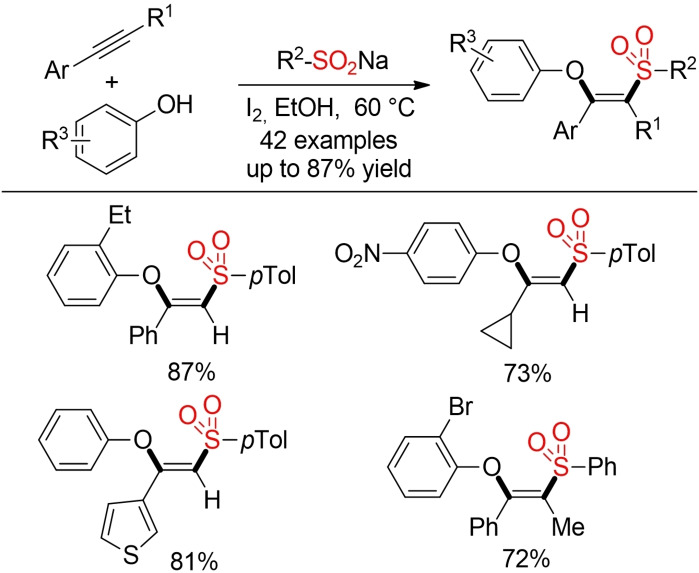
Phenoxysulfonylation of alkynes.^[59]^

In the presence of phenols as external trapping agent an efficient phenoxysulfonylation of alkynes can be achieved. Interestingly, this transformation proceeds in the absence of any transition‐metal catalyst and are solely mediated by iodine (Scheme 26).^[59]^

**Scheme 27:**
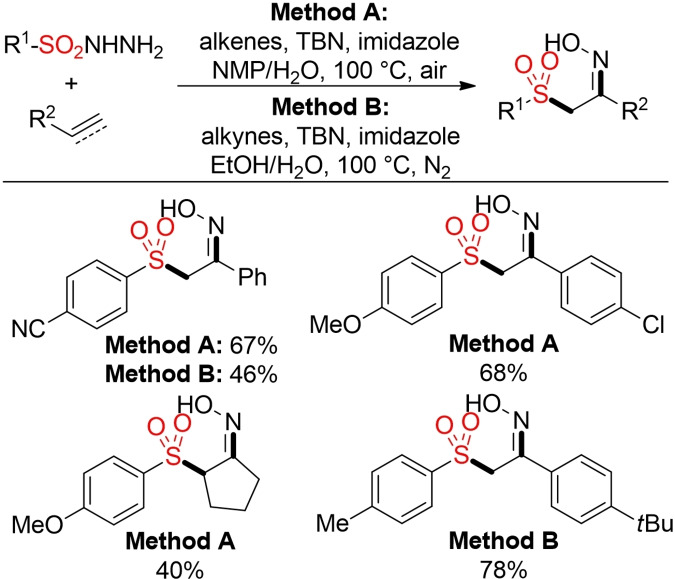
TBN‐mediated sulfonylation and oximation.^[60]^

In the presence of *tert*‐butyl nitrite (TBN) as mediator, the addition of sulfonyl hydrazides to either alkenes or alkynes leads to the formation of α‐sulfonyl ketoximes (Scheme 27).^[60]^

As all common precursors for sulfonyl radicals, such as sulfonyl chlorides or hydrazides and sulfinic acid salts, have to be prepared in additional, often step‐ and waste‐intensive processes, the direct utilization of other sulfur‐based building blocks can offer an overall more sustainable approach to the desired sulfone.

**Scheme 28:**
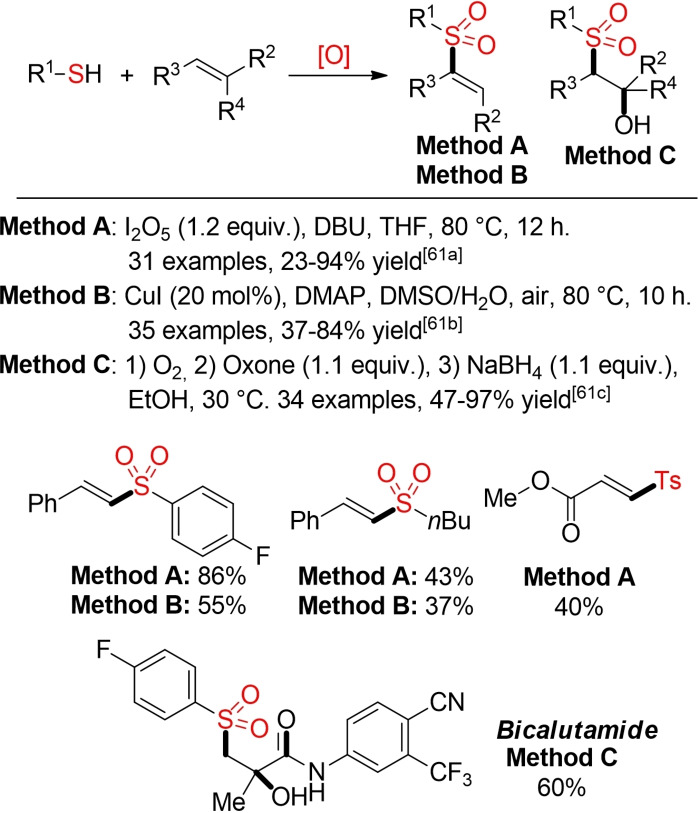
Direct utilization of thiols and alkenes.^[61]^

Thiols have been utilized for the synthesis of both vinyl sulfones and β‐hydroxysulfones from the corresponding alkenes (Scheme 28).^[61]^ Under these conditions a simultaneous oxidation of the sulfur atom and radical addition to the alkene takes place. Huo and co‐workers were able to apply their protocol to a gram‐scale synthesis of the anticancer agent bicalutamide.^[61c]^

**Scheme 29:**
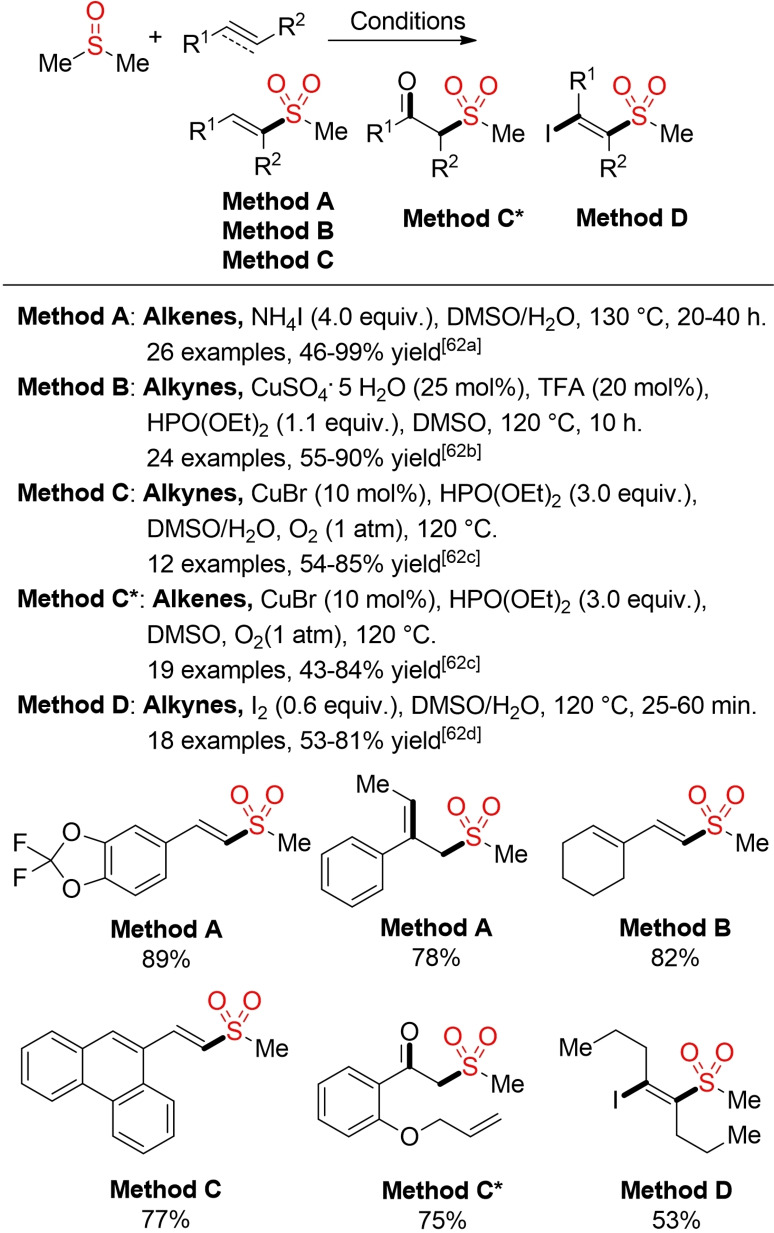
Synthesis of methyl sulfones from DMSO.^[62]^

DMSO represents a readily available and highly useful building block for the construction of methyl sulfones. Under oxidative conditions a methyl sulfonyl radical can be generated from DMSO. In turn, this radical can be trapped with alkenes and alkynes into vinyl sulfones or β‐keto sulfones. In terms of sustainability, methods utilizing water as source of the second oxygen atom are of particular interest (Scheme 29).^[62]^

One has to mention that the addition of sulfonyl radicals to alkynes or alkenes can serve as starting point for the initiation of intriguing cascade transformations towards complex (poly)cyclic scaffolds.^[63]^ Within this Review, we will not discuss the existing plethora of these transformations in detail, but rather focus on “straightforward” addition reactions.

In summary, the addition of sulfonyl radicals to alkenes and alkynes offers an atom‐economical approach not only to vinyl sulfones. However, one also has to consider the preparation of the used radical precursors (i. e., sulfonyl chlorides or sulfinic acid salts). Taking their syntheses into account, the overall sustainability profile for the synthesis of a desired sulfone might not be favorable. In this regard, the utilization of alternative radical precursors can offer an environmentally more benign pathway.

## 6 Synthesis via the Fixation of SO_2_ and SO_2_ Surrogates

**Figure 2:**
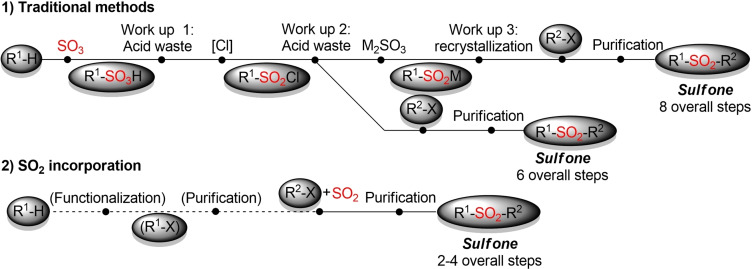
Comparison of traditional methods vs. SO_2_ incorporation.

As shown above, there has been made tremendous progress in the development of novel, more sustainable methods for the synthesis of sulfones. However, closer scrutiny reveals that all these traditional methods are hampered by the utilization of sulfur‐based building blocks. The preparation of sulfur‐containing organic molecules, such as sulfonyl chlorides and in particular sulfinic acid salts, is restricted to certain scaffolds and usually a highly waste‐ and resource‐intensive process. If one includes the synthesis of the required sulfur‐based building block, the overall sustainability profile for the production of a specific sulfone might not look favorable (Figure 2).

In the last ten years the direct incorporation of sulfur dioxide (or sulfur dioxide surrogates) into organic molecules has emerged as a versatile tool for the construction of sulfones and sulfonamides.[^9b^, ^64^] Sulfur dioxide itself is produced on an enormous annual scale and constitutes a major air pollutant. The fixation of sulfur dioxide into value‐added chemical products represents a highly attractive approach for the capture of an undesired side‐product from the combustion of fossil fuels, comparable to the fixation of carbon dioxide. One has to mention that SO_2_ is a highly toxic and corrosive gas, which complicates its safe handling in a typical laboratory setting. For small‐scale applications, this limitation can be overcome with bench‐stable and easy‐to‐handle sulfur dioxide surrogates, such as 1,4‐diazabicyclo[2.2.2]octanebis(sulfur dioxide) adduct (DABSO), introduced into organic synthesis by Deeming and Willis,^[65a]^ or different sulfite salts.^[65b]^ The introduction of such stable and safe‐to‐handle surrogates has led to tremendous advances in the construction of sulfones and sulfonamides from two sulfur‐free starting materials and SO_2_ as building block for the central SO_2_‐functionality.

**Scheme 30:**
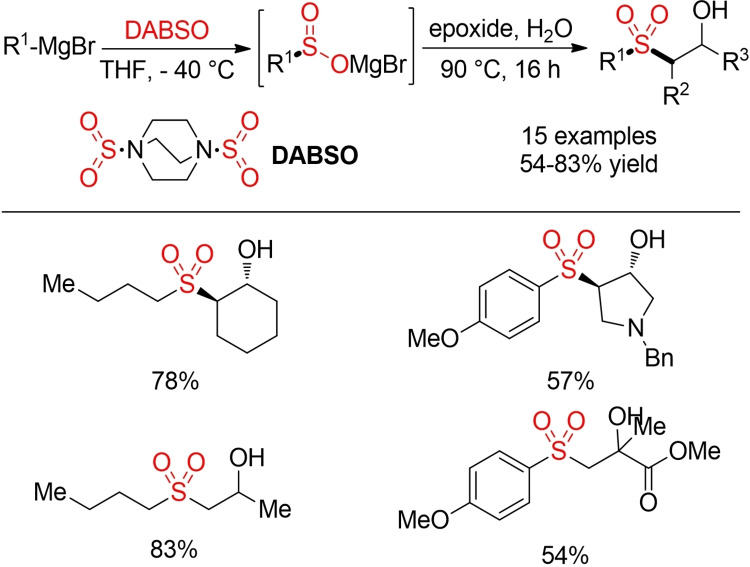
Synthesis of sulfones from organometallic compounds.^[66a]^

**Scheme 31:**
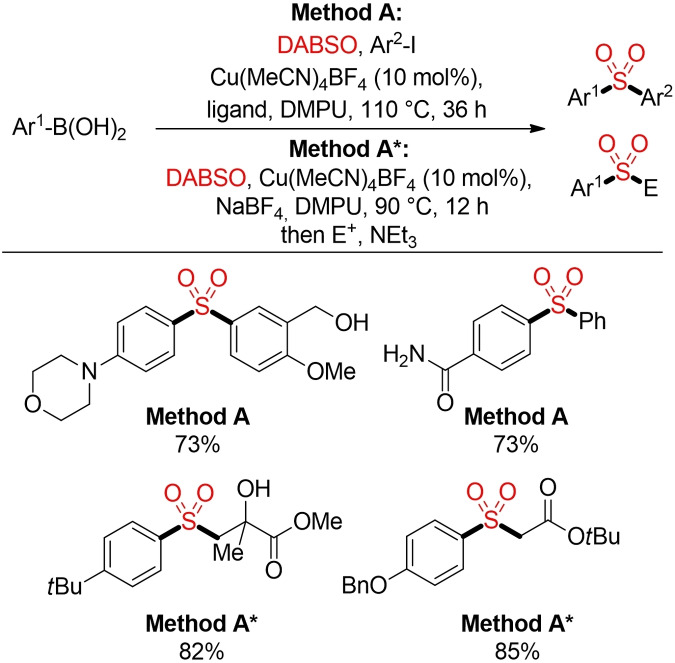
Copper(I)‐catalyzed sulfonylative Suzuki‐Miyaura cross coupling.^[66b]^

Some representative (and non‐sustainable) examples for ionic transformations exploiting nucleophilic organometallic reagents are depicted in Schemes 30 and 31. Willis and co‐workers reported a one‐pot synthesis of sulfones from Grignard and organolithium reagents via the in‐situ generation and trapping of sulfinic acid salts (Scheme 30).^[66a]^ In a similar manner, aryl sulfones can be prepared either directly in a copper‐catalyzed sulfonylative version of the Suzuki coupling or via in‐situ generated sulfinates (Scheme 31).^[66b]^

**Scheme 32:**
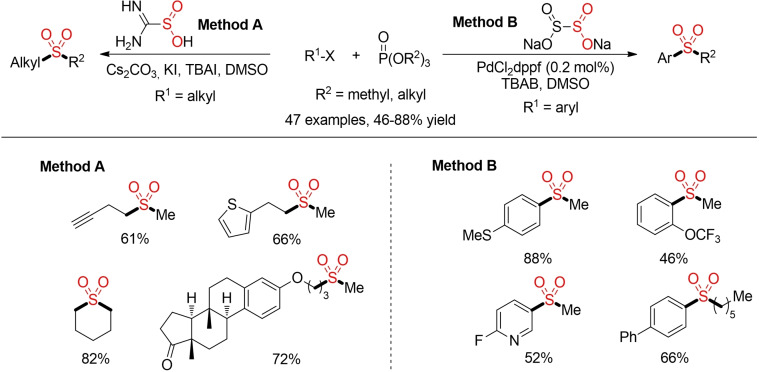
Sulfone construction via sulfur dioxide surrogate control.^[67]^

In the last years a variety of analogous methods for the in‐situ generation and trapping of sulfinate salts starting either from organometallic reagents or aryl halides in combination with a solid SO_2_ surrogate haven been reported.^[32]^ All these methods enable the direct construction of sulfones and bypass the often cumbersome, waste‐ and resource intensive separate synthesis and isolation of defined sulfur‐based building blocks (Figure 2). However, most of these reactions still rely on the use of organometallic reagents, transition metal catalysts and/or hazardous solvents. So far, the main focus is still on the exploration of novel reactivities and the incorporation of sulfur dioxide into different ionic reaction pathways. Herein we just want to highlight one more example from Jiang and co‐workers. This method provides a general approach for the construction of either alkyl alkyl or alkyl aryl sulfones from organic halides, phosphate esters and sulfur dioxide surrogates. Interestingly, the two different SO_2_ surrogates give controlled access to the desired type of sulfone. Whereas sodium dithionite in combination with low loadings of a Pd‐catalyst affords aryl alkyl sulfones, alkyl alkyl sulfones were prepared from thiourea dioxide in a catalyst‐free, already quite sustainable process (Scheme 32).^[67]^

The direct generation of sulfonyl radicals from SO_2_ (or a solid surrogate) and a suitable radical precursor offers another attractive opportunity for a more sustainable synthesis of sulfones.^[68a]^ Historically, the radical trapping of sulfur dioxide has been (and is still) frequently employed in the Meerwein and Reed syntheses of sulfonyl chlorides. However, the generated sulfonyl radicals can be utilized for the direct formation of sulfones, for example, by trapping with an alkene.^[68b]^ Based on pioneering studies from Wu and co‐workers,^[68c]^ several groups have reported different methods for the addition of in‐situ formed sulfonyl radicals to alkenes and alkynes.[^68d^, ^68e^] Typically, aryldiazonium salts are utilized as readily available, but potentially explosive radical precursors.

**Scheme 33:**
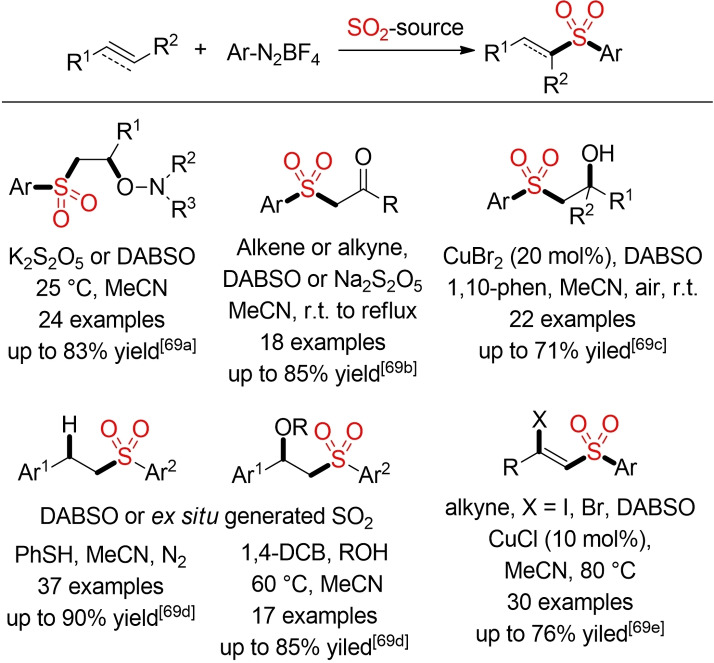
Difunctionalization of alkenes or alkynes.^[69]^

Scheme 33 contains a couple of selected examples for the vicinal difunctionalization of alkenes or alkynes utilizing in‐situ generated sulfonyl radicals generated form aryldiazonium salts and a sulfur dioxide surrogate. Usually, these three‐component reactions proceed efficiently at ambient or elevated temperatures. In the presence of an external trapping reagent (e. g., alcohol, hydroxylamine, or air), the vicinal difunctionalization can be achieved in a four‐component fashion.^[69]^ Again, most reports focus primarily on the establishment of novel reactions and reactivities. Sustainability or safety aspects (use of diazonium salts at elevated temperatures) are usually not addressed at all. Indeed, Scheme 33 only contains selected examples using acetonitrile, which is already considered as problematic solvent by the Chem21 guide. Many other examples using highly hazardous solvents, such as DCE, have been omitted.

The generation and subsequent trapping of sulfonyl radicals is a particularly valuable approach for the efficient construction of sulfonylated heterocycles.^[70]^ Unfortunately, none of the so far described methods fulfills our criteria to be considered as green alternative. Indeed, DCE is still the preeminent solvent for such transformations.

Although the construction of sulfones from sulfur dioxide and two (or more) sulfur‐free building blocks can lead to major improvements in step‐ and atom‐economy, all above mentioned methods still rely on other types of pre‐functionalized building blocks (e. g., organometallic reagents), which in turn have to be prepared in several steps. An obvious next step to enhance the overall step‐ and atom‐economy of such processes, would be the regioselective direct functionalization of a C−H bond with concomitant incorporation of sulfur dioxide. Indeed, the direct sulfonylation of C−H bonds with insertion of sulfur dioxide is an active field of research at the moment.^[71]^

**Scheme 34:**
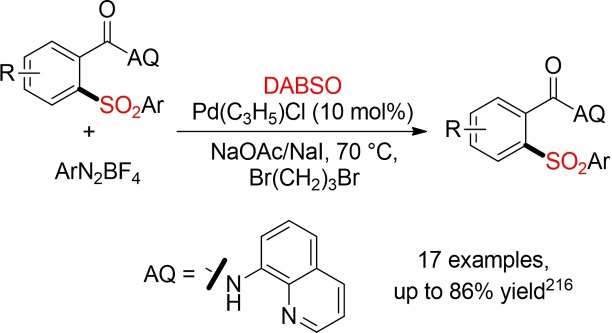
Transition‐metal‐catalyzed direct sulfonylations of C(sp^2^)−H bond with DABSO.^[72a]^

A series of new methods for palladium‐, copper‐, or cobalt‐catalyzed direct sulfonylations of C(sp^2^)−H bond with DABSO as sulfur dioxide surrogate has been disclosed in the last five years.^[72]^ All transformations exploit the directing effect of an additional functionality (directing group, DG) to achieve the regioselective functionalization of a distinct C−H bond. Overall, one could consider these types of reactions as a sulfonylative version of well‐established metal‐catalyzed C−H functionalizations. However, these initial reports are using catalysts based on rare transition metals and again hazardous organic solvents, in particular DCE. One selected example is depicted in Scheme 34. In the presence of a Pd‐catalyst a selective *ortho*‐sulfonylation of benzamide derivatives bearing an 8‐aminoqionoline DG could be achieved with DABSO and aryl diazonium salts in 1,3‐dibromopropane as problematic solvent.^[72a]^ Two further examples from Wu and co‐workers already feature some improvements towards more sustainable methods.[^72c^, ^72d^]

**Scheme 35:**
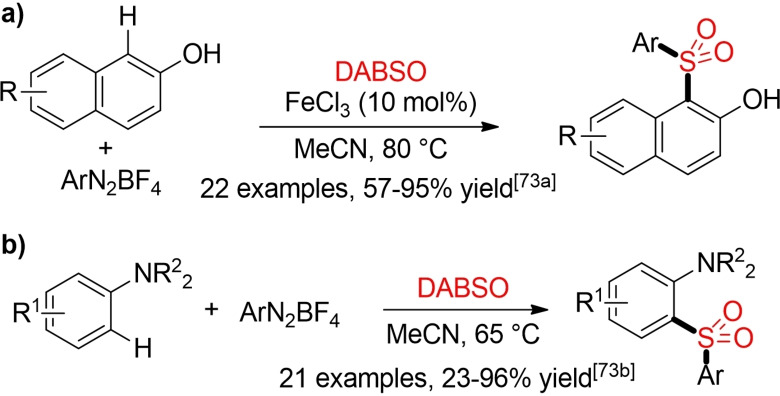
C−H sulfonylation of electron‐rich arenes.^[73]^

Using FeCl_3_ as a simple, environmentally favorable catalyst_,_ 2‐naphthols can be transformed into the desired sulfones in the presence of DABSO and aryl diazonium salts. In a similar manner, anilines can be sulfonlyated in the absence of any external catalyst (Scheme 35).^[73]^

Overall, these new developments show the tremendous potential for the development of more efficient methods for the construction of sulfones directly from sulfur dioxide. Especially the direct incorporation of SO_2_ into C−H bonds holds great promise for the development of truly sustainable processes. Yet still considerable efforts are necessary to render these initial discoveries into truly environmentally benign processes. Another point that has to be addressed is the use of sulfur dioxide surrogates. On the one hand, the use of DABSO or other SO_2_ surrogates leads to a decreased atom economy compared to the direct use of SO_2_. On the other hand, employing these stable solids as replacement for gaseous SO_2_ renders the overall process safer and minimizes the potential for a chemical accident. In our opinion, these safety benefits more than justify the use of sulfur dioxide surrogates in a typical laboratory setting. In an industrial process, the direct use of sulfur dioxide gas should be the method of choice.

## 7 Photo‐ and Electrochemical Synthesis of Sulfones

In the last twenty years novel photo‐ and electrochemical methods have emerged as powerful new tools in synthetic chemistry.^[74]^ These methods not only enable an efficient access to reactive intermediates, in particular radical species, but also provide a new, enabling technology for the development of more sustainable production processes. Using either visible‐light or electricity, preferable from renewable energy sources, as driving force for a chemical reaction can lead to more energy‐ as well as atom‐efficient chemical transformations. Therefore, it is not surprising that the photo‐ and electrochemical synthesis of sulfones has attracted considerable attention over the last years. Within the scope of this Review, we will focus primarily on methods featuring at least some aspects with importance to the development of more sustainable methods. Therefore, all reports using either photoredox‐catalysts based on rare metals (ruthenium, iridium, etc.) or hazardous solvents will not be covered.

**Scheme 36:**
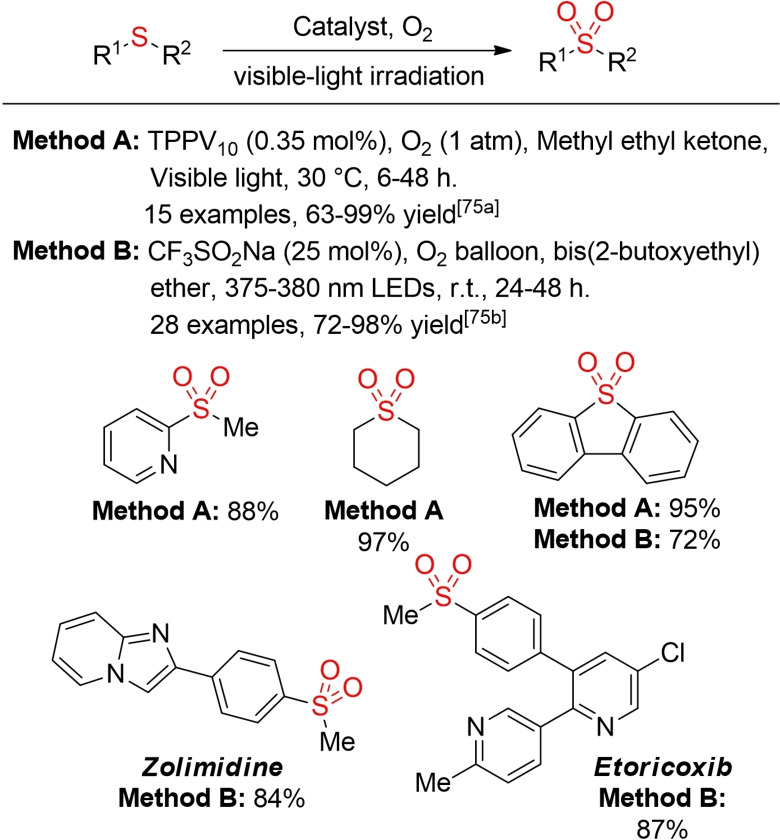
Photo‐catalyzed aerobic oxygenation of sulfides to sulfones.^[75]^

Suzuki and co‐workers described a decavanadate‐photocatalyzed oxidation of sulfides to sulfones with visible light and oxygen as terminal oxidant.^[75a]^ Interestingly, a selectivity switch between sulfoxide or sulfone formation can be realized by the choice of cosolvents. Another report utilizes a combination of CF_3_SO_2_Na and 2‐butoxyethyl ether to promote a visible‐light mediated selective oxidation towards sulfones or sulfoxides, again with oxygen as terminal oxidant (Scheme 36).^[75b]^

**Scheme 37:**
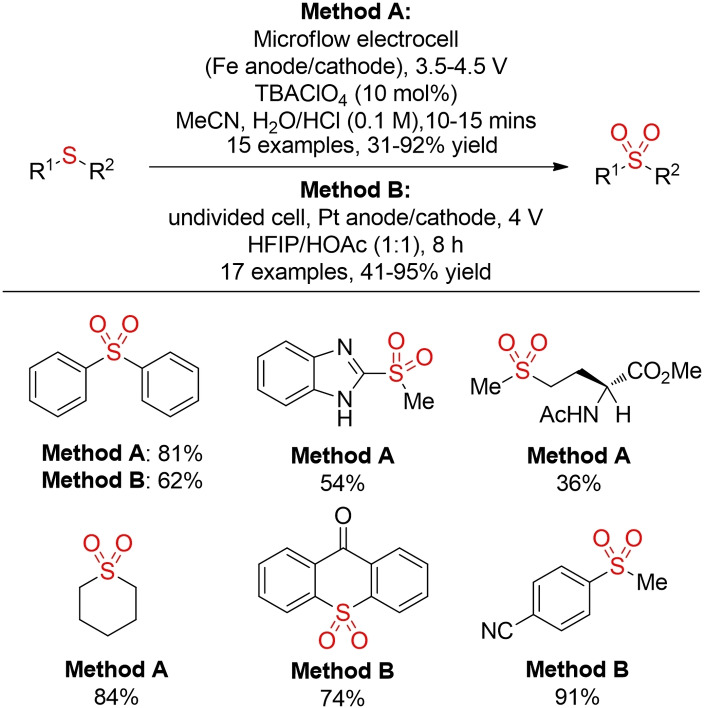
Electrochemical oxidation of sulfides.^[76]^

Another interesting recent development is the electrochemical oxidation of sulfides under flow conditions reported by Noël and co‐workers.^[76a]^ In this process water serves as the oxygen source and the chemoselectivity of the reaction can be controlled by the applied potential: whereas a lower potential leads to the sulfoxide, sulfones can be generated with higher potentials. A similar observation has been reported by Xu and co‐workers in a batch setup using HFIP as a solvent (Scheme 37).^[76b]^

Photo‐ and electrochemical methods provide a highly facile entry into the field of sulfonyl radicals.^[77]^ The generation of sulfonyl radicals mediated by visible‐light or electricity and their and subsequent addition to alkenes or alkynes has received particular attention.

**Scheme 38:**
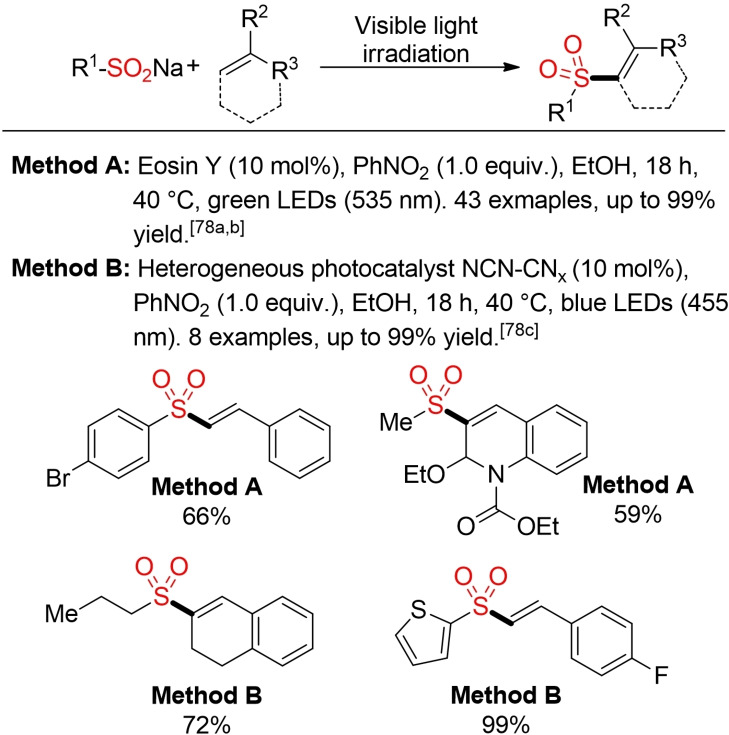
Visible‐light mediated synthesis of vinyl sulfones.^[78]^

Inspired by the pioneering work from König and co‐workers (Scheme 38),^[78]^ various protocols for the visible‐light mediated addition of different sulfonyl precursors to alkenes to construct vinyl sulfones have been reported in the last years^[79]^ Interestingly, the original report form König and co‐workers already features some important aspects of sustainability, for example, the use of eosin as a metal‐free photoredox‐catalyst and EtOH as benign solvent. Surprisingly, all subsequently reported methods contain at least one element of concern from a sustainability perspective (hazardous solvent, rare metal‐catalyst). Later on, Meyer et al. described an improved process utilizing a heterogenous, recyclable photocatalyst.^[78c]^

**Scheme 39:**
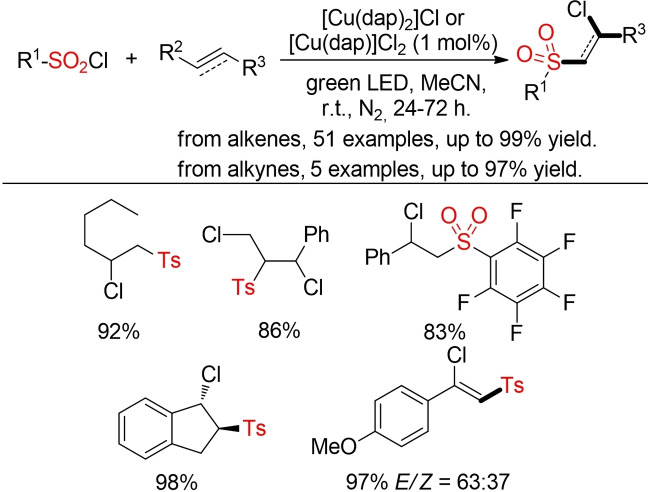
Chlorosulfonylation of alkenes and alkynes.^[80]^

Reiser and co‐workers described complementary copper‐catalyzed visible‐light mediated chlorosulfonylation of alkenes and alkynes with sulfonyl chlorides (Scheme 39).^[80]^

Although analogous methods for the direct hydrosulfonylation of alkenes or alkynes via photoredox‐catalyzed processes have been reported, none of these processes fulfill our criteria for this Review.^[81]^

**Scheme 40:**
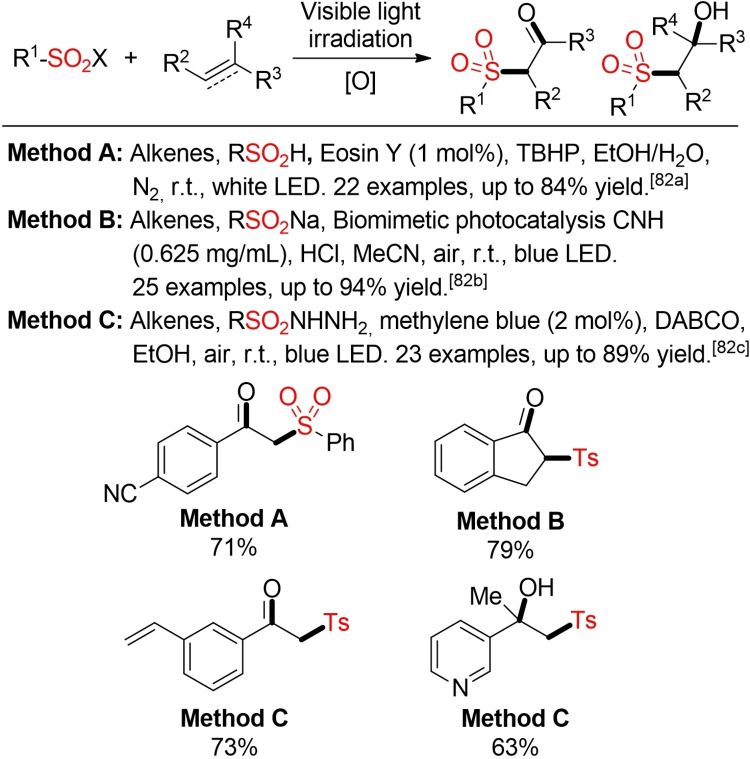
Oxysulfonylation of alkens or alkynes.[^82a^, ^82b^, ^82c^]

Several groups have described visible‐light‐promoted oxysulfonylation reactions of alkenes or alkynes with sulfinates and sulfinic acids, sulfonyl chlorides, or sulfonyl hydrazides. Depending on the used substrate, the terminal oxidant, and the reaction conditions either β‐hydroxy sulfones or β‐keto sulfones can be accessed.^[82]^ In Scheme 40 some selected examples utilizing organic dye‐based photoredox‐catalysts and solvents recommend by the Chem21 guidelines and with acetonitrile are depicted.

**Scheme 41:**
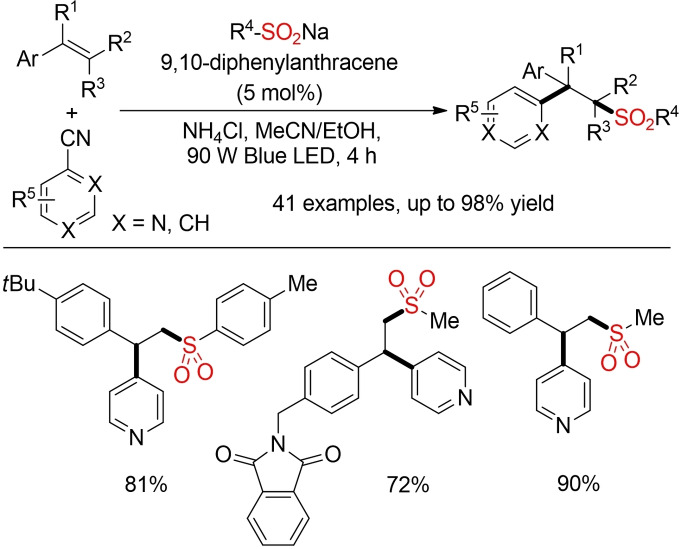
Three‐component pyridylation‐sulfonylation of styrenes.^[83b]^

Opatz and co‐workers as well as Chu and co‐workers described two complementary approaches for a photo‐induced tandem pyridylation‐sulfonylation of styrenes. This three‐component reaction is based on the trapping of a sulfonyl radical with an alkene followed by the interception of the newly formed carbon radical with pyridyl radical anion species. Whereas the approach of Opatz and co‐workers (not shown) is based on an Ir‐catalyst,^[83a]^ the method of Chu and co‐workers utilizes a simple organic dye in a MeCN/EtOH solvent mixture (Scheme 41).^[83b]^

**Scheme 42:**
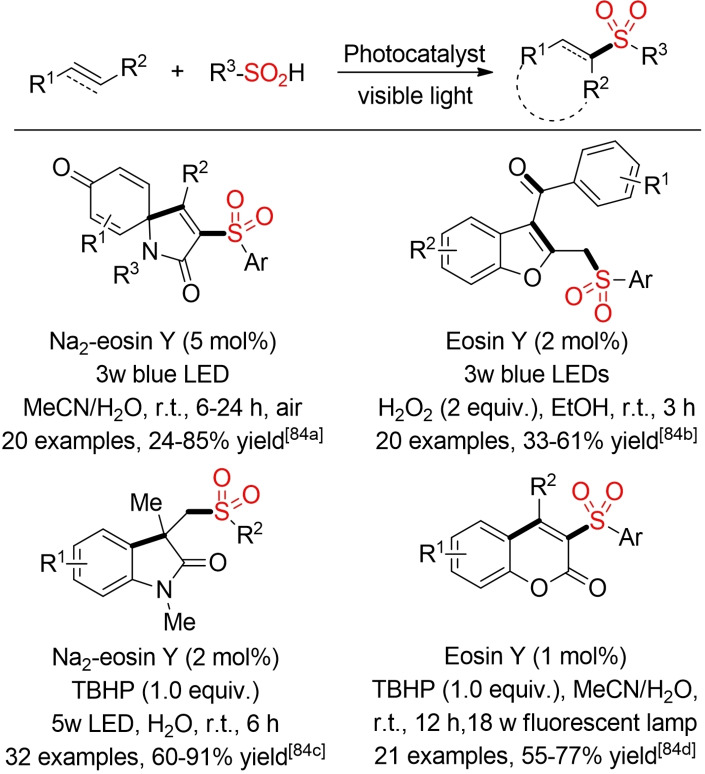
Visible‐light‐induced cyclization via sulfonyl‐radicals.^[84]^

As in the case of classical sulfonyl‐radical chemistry, the photoredox‐catalyzed generation of such radicals has been exploited for the synthesis of various heterocycles via sulfonyl‐radical induced cyclization reactions. Some selected examples are depicted in Scheme 42.^[84]^

**Scheme 43:**
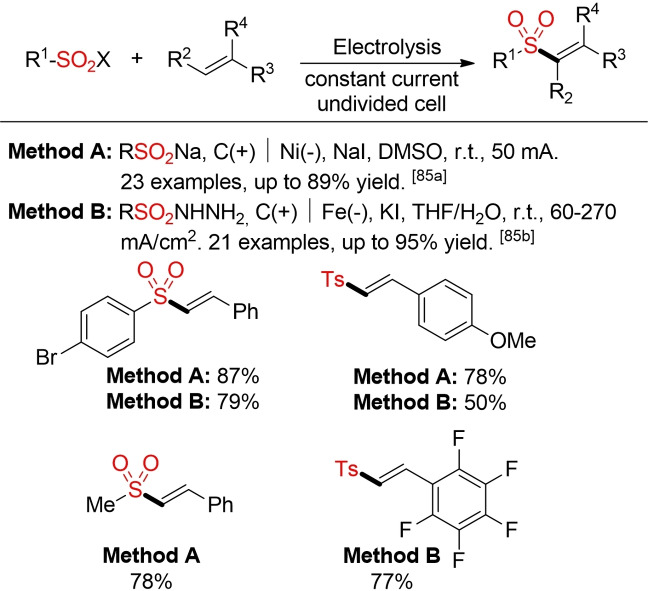
Electrochemical synthesis of vinyl sulfones.^[85]^

The vinyl sulfone scaffold can be accessed in a complementary manner via the electrochemical coupling of alkenes with sulfinate salts or sulfonyl hydrazides. In both reports, iodide salts serve as promoters and supporting electrolytes (Scheme 43).^[85]^

**Scheme 44:**
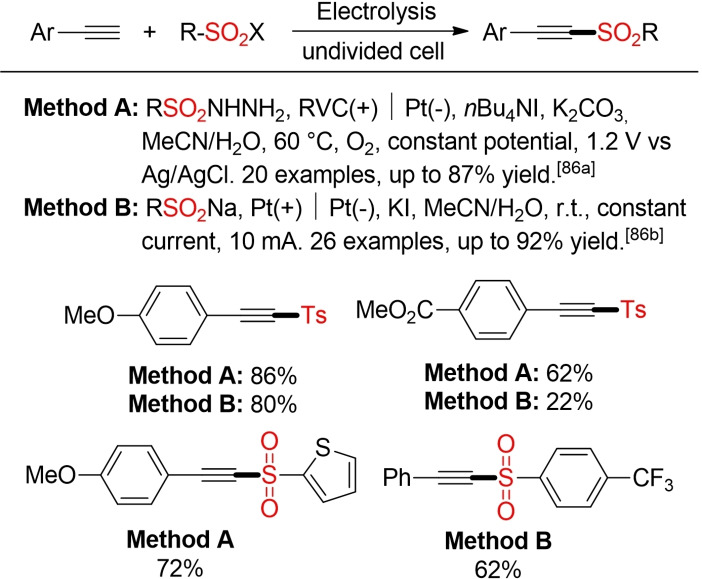
Electrochemical synthesis of vinyl sulfones.^[86]^

In a similar way, alkynyl sulfones can be prepared via an electrochemical coupling of terminal alkynes with either sulfinic acid salts or sulfonyl hydrazides (Scheme 44). Again, the addition of an iodide salt is crucial to achieve an efficient transformation.^[86]^

**Scheme 45:**
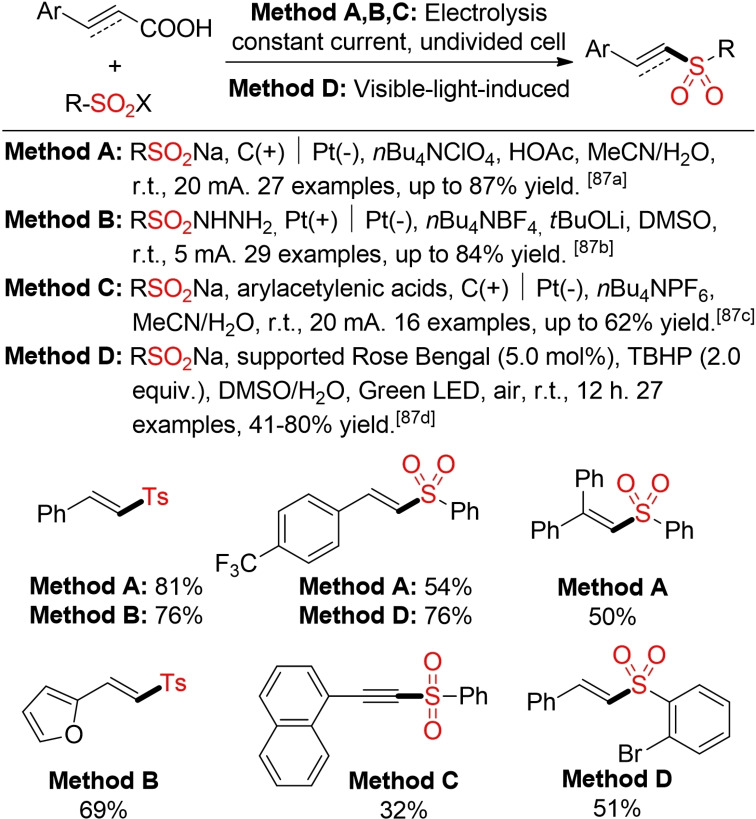
Decarboxylative synthesis of vinyl and alkynyl sulfones.^[87]^

The decarboxylative electrochemical or photochemical coupling of cinnamic or aryl acetylenic acids with sodium sulfinates or sulfonyl hydrazides offers an alternative method for the construction of alkenyl and alkynyl sulfones (Scheme 45).^[87]^

**Scheme 46:**
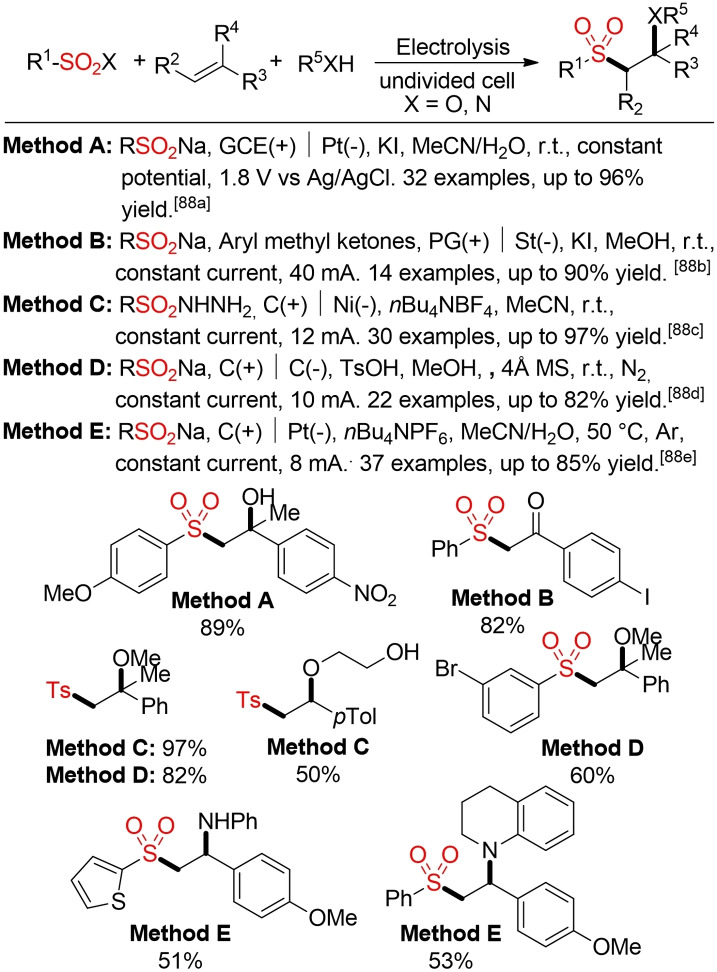
Electrochemical difunctionalization of alkenes.^[88]^

The electrochemical oxidative sulfonylation of alkenes using either sulfinic acid salts or sulfonyl hydrazides as radical precursors gives access to β‐alkoxy or β‐keto sulfones in the absence of an external oxidant (Scheme 46).^[88]^ The electrochemical difunctionalization of alkenes was extened to the construction β‐amino sulfones by Li and co‐workers.^[88e]^

**Scheme 47:**
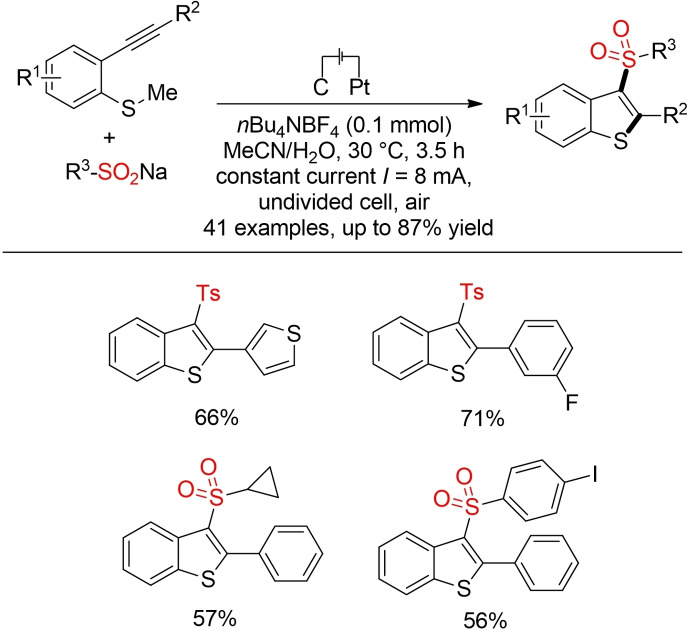
Synthesis of sulfonated benzothiophenes via electrooxidative tandem cyclization.^[89]^

The elctrochemical generation of sulfonyl radicals has also been harnessed for the construction of heterocycles, such as sulfonylated benzothiophens (Scheme 47).^[89]^ In comparison to their photoredox‐catalyzed counterparts, electrochemical methods usually do not rely extensively on hazadorous solvents.

The photo‐ or electrochemical direct sulfonylation of C−H bonds offers an attractive opportunity for the development of more sustainable sulfonylation procedures.

**Scheme 48:**
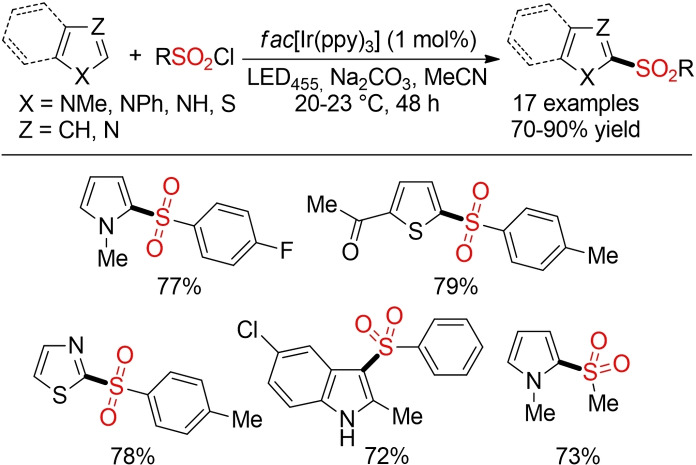
Visible‐light‐induced photocatalytic sulfonylation of heteroarenes.^[90]^

Reiser and co‐workers reported a method for the direct sulfonylation of heteroarenes mediated by visible light (Scheme 48). Interestingly, the outcome of this reaction can be controlled by modulation of the temperature. Whereas a reaction at ambient temperature affords the sulfone product, SO_2_ extrusion is observed at higher temperatures. Of course, the use of an iridium‐based catalyst represents a significant disadvantage, at least from the viewpoint of sustainability. However, this report showcases that visible‐light‐mediated photo(redox) catalysis can provide an attractive opportunity for the development of more sustainable sulfone syntheses.^[90]^

**Scheme 49:**
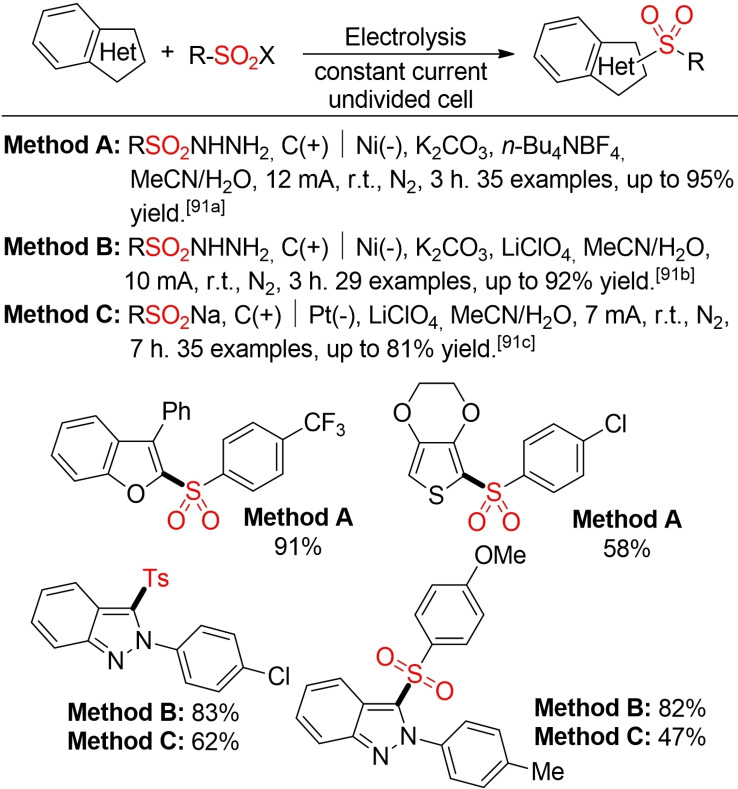
Electrochemical sulfonylation of five‐membered heterocycles.^[91]^

Oh and co‐workers, Lei and co‐workers, and De Sarkar and co‐workers have reported three methods for the electrochemical sulfonylation of different heterocycles (Scheme 49). With the concomitant production of H_2_ instead of employing a stoichiometric co‐oxidant, these methods offer an alternative pathway towards a more sustainable sulfone synthesis.^[91]^

**Scheme 50:**
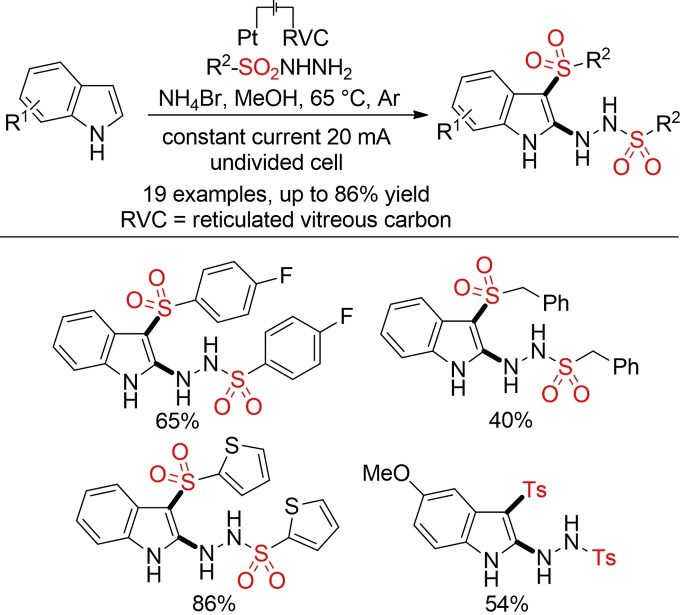
Electrochemical sulfonylation of indoles.^[92]^

Interestingly, the electrochemical sulfonylation of indoles with sulfonyl hydrazides leads to a simultaneous hydrazination at the C2 position (Scheme 50).^[92]^

**Scheme 51:**
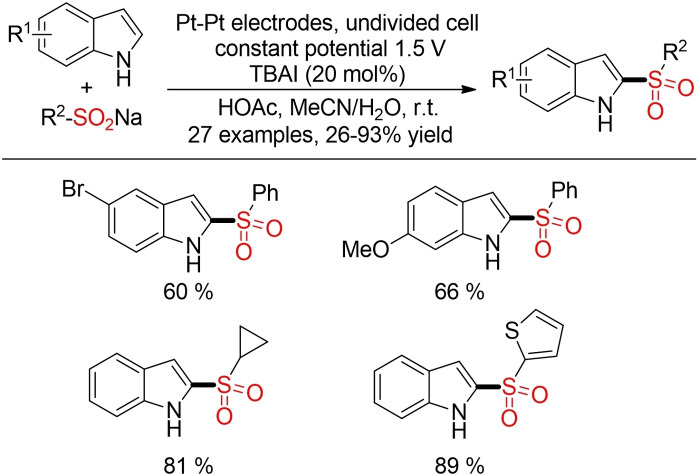
Electro‐oxidative sulfonylation of indoles.^[93]^

On the other hand, by using sulfinates as building block, Yu and co‐workers could achieve a regioselective, electrochemical sulfonylation of indoles (Scheme 51).^[93]^ In this case, reactive I_2_ is (re)generated by electricity.

**Scheme 52:**
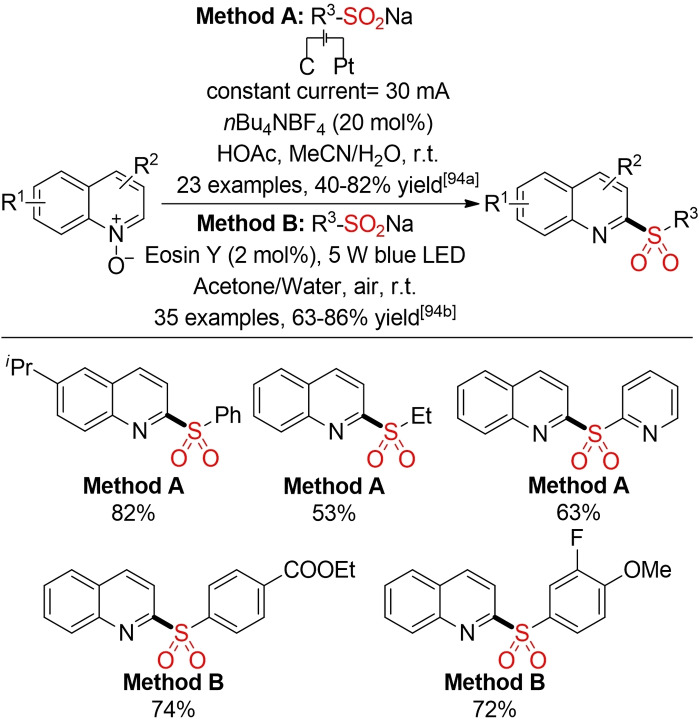
Deoxygenative C2 sulfonylation of quinoline *N*‐oxides with sulfinates.^[94]^

Lei and co‐workers and He and co‐workers were able to introduce electrochemical and photoredox‐catalyzed methods for the deoxygenative C2 sulfonylation of quinoline *N*‐oxides with sulfinic acids or their salts respectively (Scheme 52).^[94]^ In both cases, the substrate itself serves as a terminal oxidant. By employing either electricity or an excited state photoredox‐catalyst, these methods avoid the use of high temperatures or stoichiometric amounts of co‐oxidants, as described in prior reports.^[95]^

**Scheme 53:**
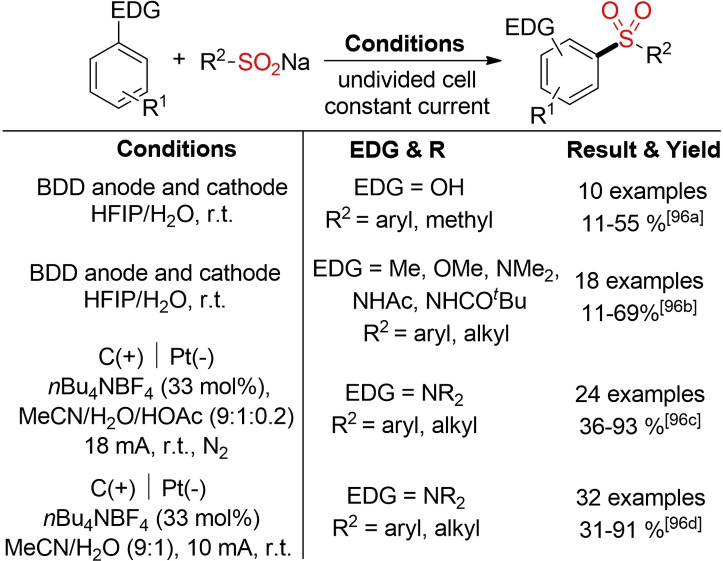
Direct electrochemical C−H sulfonylation of electron‐rich arenes.^[96]^

In the last few years, analogous methods for a direct electrochemical sulfonylation of electron‐rich arenes, such as phenols or anilines, were described by several groups (Scheme 53). In general, these electrochemical, direct C−H sulfonylation reactions provide an attractive opportunity for a reagent‐free and therefore more sustainable sulfone synthesis.^[96]^ From an environmental standpoint, the use of hexafluoroisopropanol (HFIP) should be regarded carefully. However, it has been shown that HFIP can be an excellent electrolyte for electrochemical transformations.^[97]^

Both photo‐ and electrochemical methods offer an attractive approach for the generation of various reactive radical species via single‐electron transfer transformations. In combination with sulfur dioxide as highly radicalophilic species, this can lead towards more sustainable processes for the direct fixation of sulfur dioxide using visible light or electricity from renewable energy sources.^[98]^

In the last years Wu and co‐workers have pioneered photoredox‐catalyzed sulfur dioxide insertion reactions based on in‐situ generated radical species.^[99]^ Within this Review, a few methods utilizing different radical precursors in combination with organic dyes as catalyst and non‐hazardous solvents will be highlighted.

**Scheme 54:**
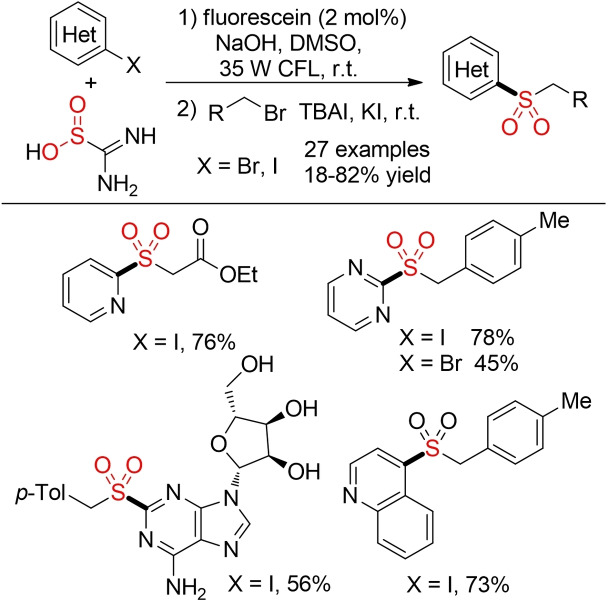
Photoredox sulfonylation with thiourea dioxide.^[100]^

In the presence of the organic dye fluorescein as catalyst, aryl alkyl sulfones can be prepared in good yields using thiourea dioxide as SO_2_ surrogate (Scheme 54). This method provides an attractive alternative to similar methods exploiting organometallic nucleophiles. However, this method is so far limited to activated (hetero)aryliodides.^[100]^

**Scheme 55:**
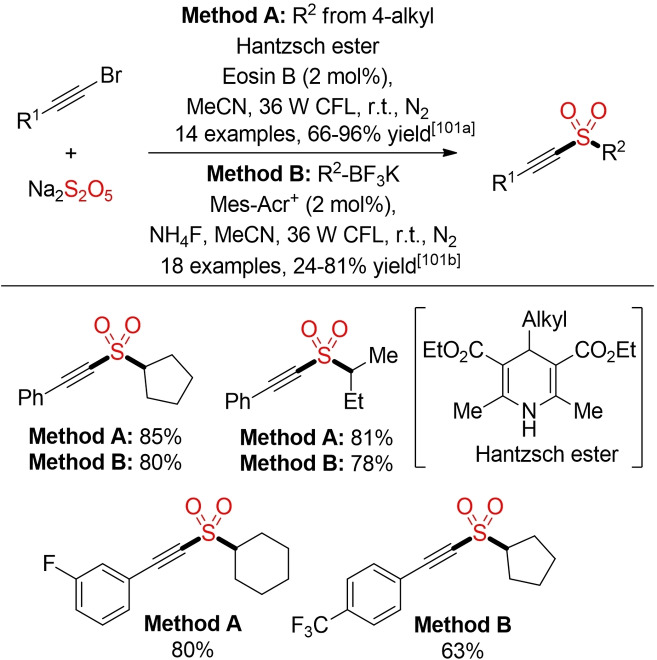
Photoredox three‐component synthesis of alkynyl sulfones.^[101]^

Starting from Hantzsch esters or alkyl trifluoroborates as radical precursors, alkynyl sulfones can be prepared via a visible‐light mediated coupling with alkynyl bromides in the presence of Na_2_S_2_O_5_ as SO_2_ surrogate and an organic dye as catalyst (Scheme 55).^[101]^

**Scheme 56:**
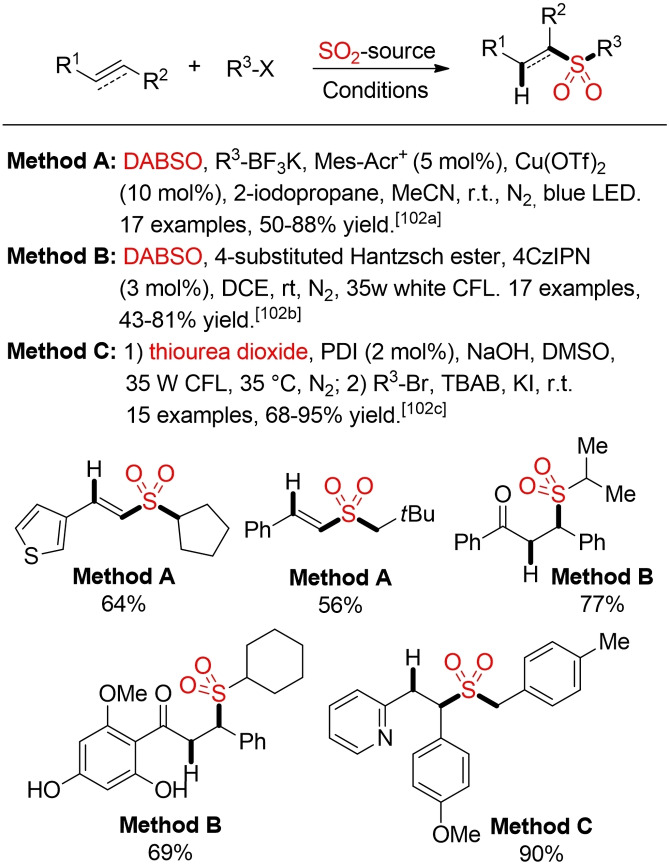
Photoredox‐catalyzed hydrosulfonylation of alkenes and alkynes.^[102]^

The visible‐light mediated generation of sulfonyl radicals from suitable precursors and a SO_2_ source offers an attractive opportunity for the sulfonylation of alkenes and alkynes. Using either alkyl trifluoroborates or 4‐substituted Hantzsch ester Wu and co‐workers described several approaches for the hydrosulfonylation of alkenes and alkynes via a photocatalytic fixation of SO_2_ (Scheme 56).^[102]^ While proceeding through a different mechanistic pathway, the photoredox‐catalyzed functionalization of alkenes with thiourea dioxide gives access to the same type of alkyl sulfone products (Scheme 56, method C).^[102c]^

**Scheme 57:**
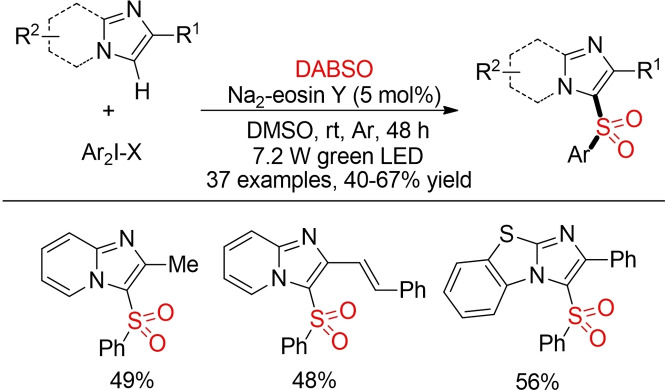
Photoredox‐catalyzed C−H‐sulfonylation of imidazoheterocycles.^[103]^

Piguel and co‐workers described an eosin‐catalyzed direct C−H‐sulfonyation of imidazoheterocycles using diaryliodonium salts and DABSO (Scheme 57).^[103]^ The utilization of diaryliodonium salts results in an overall decreased atom‐economy. On the other hand, this method offers an intriguing opportunity for the direct functionalization of single C−H bonds with SO_2_.

**Scheme 58:**
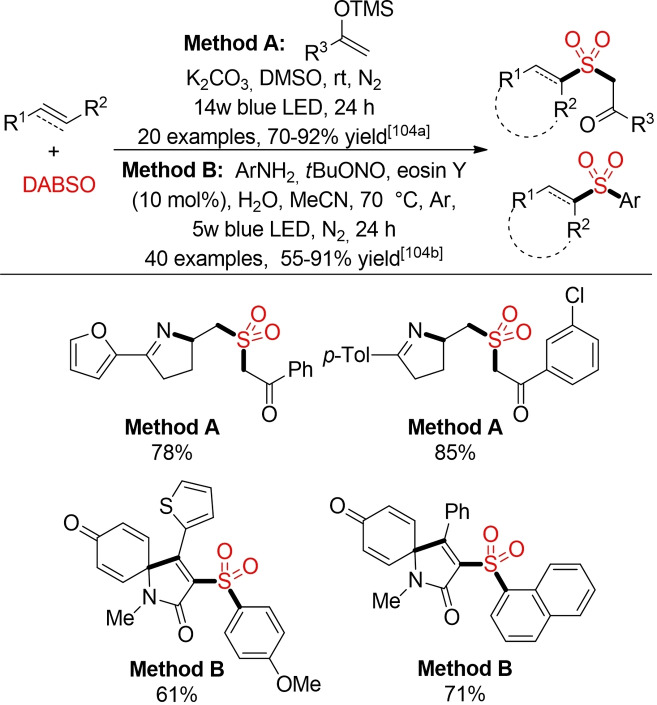
Photoredox‐catalyzed three‐component sulfonylation.^[104]^

Wu and co‐workers and Tang and co‐workers reported novel photoredox‐catalyzed cyclization cascades initiated by in‐situ generated sulfonyl radicals for the construction of sulfonylated nitrogen‐containing heterocycles (Scheme 58).^[104]^

**Scheme 59:**
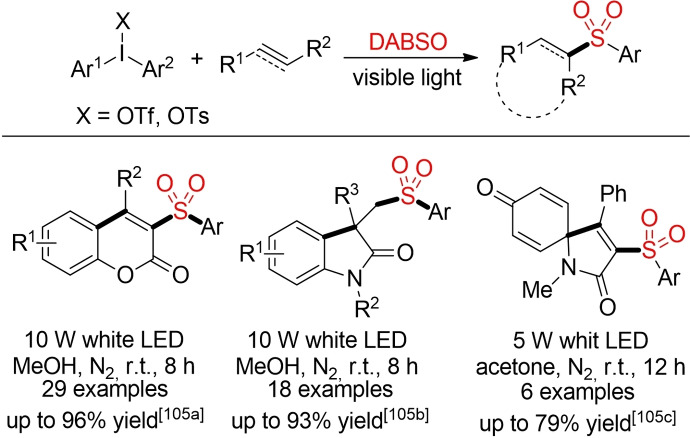
Visible‐light mediated sulfonylation with diaryliodonium salts.^[105]^

Manolikakes and co‐workers and Volla and co‐workers reported the direct, visible‐light mediated insertion of sulfur dioxide into sulfonylated coumarins, oxindoles and azaspiro[4,5]‐trienones using diaryliodonium salts (Scheme 59). Although the utilization of diaryliodonium salts does lead to an unfavorable atom economy, these processes demonstrate the feasibility of a direct fixation of sulfur dioxide, a common air pollutant solely driven by visible light (e. g., by irradiation with sunlight) in the absence of an external catalyst or sensitizer.^[105]^

Overall, photo‐ and electrochemical techniques provide highly enabling tools for a more sustainable construction of sulfones. Apart from replacing stoichiometric reagents with visible light or electricity as driving force, these methods provide ample opportunities to explore novel types of reactivities and access so far inaccessible scaffolds. In combination with the direct fixation of sulfur dioxide, photo‐ and electrochemical methods hold a unique position for groundbreaking developments in the field of sustainable sulfone synthesis. Whereas the photochemical fixation of sulfur dioxide already has received considerable attention, electrochemical counterparts are still missing.

## 8 Conclusion and Perspective

Sulfones constitute a privileged class of organic molecules with numerous applications in various areas. Considerable efforts have been devoted towards the development of new and improved methods for a more sustainable synthesis of this important functional group. In the last years tremendous progress has been achieved in different areas. On the one hand, traditional methods for the synthesis of sulfones haven been improved in terms of their overall sustainability profile. The introduction of more efficient catalysts or less harmful solvents or oxidants has led to “greener” versions of these classical processes. Despite these advances, all classical methods share an inherent disadvantage, the utilization of pre‐functionalized, sulfur‐containing starting materials. Even an almost ideal sulfone synthesis based on such building blocks, such as the oxidation of sulfides with aq. H_2_O_2_, will in turn require the synthesis of the corresponding starting materials in additional, often waste‐ and resource‐intensive processes. These shortcomings have been addressed by developments in two other areas, the selective functionalization of C−H bonds and the controlled introduction of the sulfonyl functionality using sulfur dioxide or suitable surrogates. One has to mention that both approaches are still in their infancy (as compared to the classical methods) and research has been mostly devoted towards the identification and development of novel reactions and reactivity concepts. Important aspects of sustainability, such as solvents, the use of stoichiometric, metal‐based co‐oxidants, harmful reagents, and others, still have to be addressed in the future. Indeed, we had to dismiss a plethora of reports from these fields for our Review due to their highly unfavorable environmental profile. Just to point out one single, overarching problem within most of these methods, the highly hazardous solvent 1,2‐dichloroethane (DCE) is the most preeminent solvent in C−H sulfonyation reactions and photoredox‐catalyzed transformations. Replacing DCE with environmental more benign solvents would lead to a significant advance in terms of sustainability. Still, the combination of selective C−H functionalization with concomitant SO_2_ insertion can provide the fundamental base towards the implementation of a truly sustainable synthesis of sulfones. Insights and developments in the fields of photo‐ and electrochemistry will provide another driving force for the development of more sustainable methods.

To sum it up, significant advances towards the “greener” synthesis of sulfones haven been achieved. Still, it will be a long way to go towards a truly sustainable, “ideal” synthesis, but new paths are open and the journey will be an exciting one.

## Conflict of interest

The authors declare no conflict of interest.

## Biographical Information

*Shuai Liang was born in Qingdao (P. R. of China) in 1988. He earned his BS and MS degree in 2011 and 2014 from Sichuan University, under the guidance of Prof. Xiaoqi Yu. In 2018 he obtained his PhD in organic chemistry from Goethe‐University Frankfurt, under the supervision of Prof. Dr. Georg Manolikakes. Since 2019, he joined the School of Pharmacy, Qingdao university as an assistant professor. His current research interest focuses on synthetic methodology studies of sulfonyl‐group containing compounds and their applications in medicinal chemistry*.

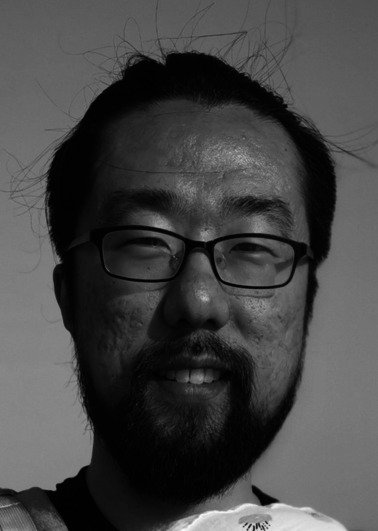

## Biographical Information

*Georg Manolikakes studied chemistry at the Ludwig‐Maximilians University (LMU) Munich. At the LMU he joined the group of Prof. Paul Knochel and received his PhD in 2009 in the field of organometallic chemistry. After a postdoctoral stay with Prof. Phil S. Baran at the Scripps Research Institute, he started his independent career at the Goethe‐University Frankfurt in 2010. In 2017, he was appointed as associate professor at the Technical University Kaiserslautern. His research interests cover multi‐component and one‐pot reactions, the synthesis of sulfonyl‐containing molecules, asymmetric synthesis, and medicinal chemistry*.